# Similar striatal gene expression profiles in the striatum of the YAC128 and HdhQ150 mouse models of Huntington’s disease are not reflected in mutant Huntingtin inclusion prevalence

**DOI:** 10.1186/s12864-015-2251-4

**Published:** 2015-12-21

**Authors:** Zubeyde Bayram-Weston, Timothy C. Stone, Peter Giles, Linda Elliston, Nari Janghra, Gemma V. Higgs, Peter A. Holmans, Stephen B. Dunnett, Simon P. Brooks, Lesley Jones

**Affiliations:** MRC Centre for Neuropsychiatric Genetics and Genomics, School of Medicine, Cardiff University, Cardiff, CF24 4HQ UK; Brain Research Group, School of Bioscience, Cardiff University, Cardiff, CF10 4AX UK

**Keywords:** Huntington’s disease, Neurodegeneration, Gene expression, Transgenic mouse models, Behaviour

## Abstract

**Background:**

The YAC128 model of Huntington’s disease (HD) shows substantial deficits in motor, learning and memory tasks and alterations in its transcriptional profile. We examined the changes in the transcriptional profile in the YAC128 mouse model of HD at 6, 12 and 18 months and compared these with those seen in other models and human HD caudate.

**Results:**

Differential gene expression by genotype showed that genes related to neuronal function, projection outgrowth and cell adhesion were altered in expression. A Time-course ANOVA revealed that genes downregulated with increased age in wild-type striata were likely to be downregulated in the YAC128 striata. There was a substantial overlap of concordant gene expression changes in the YAC128 striata compared with those in human HD brain. Changes in gene expression over time showed fewer striatal YAC128 RNAs altered in abundance than in the HdhQ150 striata but there was a very marked overlap in transcriptional changes at all time points. Despite the similarities in striatal expression changes at 18 months the HdhQ150 mice showed widespread mHTT and ubiquitin positive inclusion staining in the striatum whereas this was absent in the YAC128 striatum.

**Conclusions:**

The gene expression changes in YAC128 striata show a very closely matched profile to that of HdhQ150 striata and are already significantly different between genotypes by six months of age, implying that the temporal molecular gene expression profiles of these models match very closely, despite differences in the prevalence of brain inclusion formation between the models. The YAC128 gene expression changes appear to correlate well with gene expression differences caused by ageing. A relatively small number of genes showed significant differences in expression between the striata of the two models and these could explain some of the phenotypic differences between the models.

**Electronic supplementary material:**

The online version of this article (doi:10.1186/s12864-015-2251-4) contains supplementary material, which is available to authorized users.

## Background

Huntington’s disease (HD) is a fatal progressive neurodegeneration with motor, cognitive and psychiatric manifestations. It is caused by an expansion of a CAG triplet repeat in exon 1 of the *HTT* gene, which is translated to give an expanded glutamine tract at the N-terminus of the protein, huntingtin (HTT) [1]. A series of genetic mouse models of the disease have been generated using various technologies to give transgenic and knock in models of the disease that include both truncated and full-length *Htt* [2–8]. These models have been tested in multiple behavioural paradigms and show deficits in tests of motor ability and in cognitive and behavioural assessments [5, 9–16].

Changes in gene expression have also been seen in the brains and other tissues of the mouse models of HD. These changes show a substantial overlap between the various mouse models tested and also overlap with gene expression changes seen in human HD brain [17]. The profiles of genes with reduced expression appear to overlap to greater extent between models and between models and human brain than those with increased expression [17, 18]. Treatments that alleviate the decreased expression can improve the phenotype in mouse models, whether directed at transcriptional mechanisms or not [19–23].

The YAC128 model of HD carries a full length human *HTT* gene in a GSE70656 artificial chromosome, and extensive behavioural testing has shown that it displays substantial phenotypes that correlate with human HD symptoms from a relatively early age [13, 14, 16, 24, 25] including affective symptoms [26]. Deficits on the rotarod were observed from 4 months of age and persisted throughout life, and deficits on the balance beam occurred from 8 months [27]. In the water maze, reduced ability to find the hidden platform was seen at 8–10 months with reversal learning showing deficits from 4 months [27]. Deficits in reversal learning in a set-shifting task were seen by 6 months and of extra-dimensional set-shifting at 16 months [28], although no implicit learning deficits were observed in these mice [29]. Despite these early changes in the behavioural phenotype of the mice, frank neuronal inclusions were only visible from 15 months of age, although diffuse staining with antibodies that detect inclusions was seen from 12 months of age [30].

As HD affects the caudate and putamen earliest and most profoundly and shows substantial gene expression dysregulation [18] we chose to examine mouse striatum, as the nearest equivalent, in order to investigate whether dysregulated gene expression also occurred in the YAC128 striatum. We examined global gene expression changes in the striata from wild-type (WT) and transgenic mice from the YAC128 mouse line at 6, 12 and 18 months. We observed alterations in gene expression at all time points, which overlapped with changes seen in human HD brain and in other mouse models of the disease. We observed that genes downregulated with age in normal mice tended to be even more decreased in expression in the YAC128 striata.

## Results

### The effects of age on gene expression

Between 6 and 12 months, 2469 mRNAs (probesets: 1272 distinct genes) are altered in abundance in the WT animals and 1266 mRNAs (800 genes) in the YAC128 animals (nominal *p* <0.05). Of these, 241 mRNAs (156 genes), more than expected by chance, occur in both sets (9.8 % and 19.0 % of probesets respectively, *p* = 0.045). Between 12 and 18 months, more mRNAs are altered: 2579 mRNA 1352 genes) in WT mice and 3019 (2211 genes) in YAC128 mice, of which 522 mRNA (398 genes) are common to both cohorts (20.2 % and 17.3 % of probesets respectively, *p* <10^−4^). The overlapping probesets and corresponding genes are given in Additional file 1: Table S1. A GO term enrichment analysis (Table 1, Additional file 2: Table S2) shows that few pathways show an over-representation of significantly differentially expressed genes: only translation (GO:0006412) in the WT 6–12 month striata and cell adhesion (GO:0007155) in the YAC128 6–12 month striata are close to significance. Translation does not appear as even nominally significant in the YAC128 data, nor cell adhesion in the WT data (Additional file 2: Table S2). Between 12 – 18 months several processes are significantly over-represented in both WT and YAC128 striata (Table 1), including neuronal processes in both cohorts. Cell adhesion (GO:0007155) is significantly over-represented in the YAC128 striata over this time period.

**Table 1:** Pathways altered with age in mouse striatum in YAC128 mice

ID	GO term	*p*-value	FDR p	Count	Global
WT 6 - 12 m					
GO:0006412	Translation	2.00E-05	0.043	39	317
HD 6 - 12 m					
GO:0007155	Cell adhesion	3.03E-05	0.059	32	384
WT 12 - 18 m					
GO:0007608	Sensory perception of smell	2.47E-21	2.07E-18	68	1098
GO:0007600	Sensory perception	1.80E-17	1.51E-14	70	1364
GO:0007186	G-protein coupled receptor protein signaling pathway	1.64E-16	1.37E-13	76	1657
GO:0050877	Neurological system process	1.17E-14	9.73E-12	72	1620
GO:0007165	Signal transduction	2.03E-09	1.69E-06	100	3381
GO:0019236	Response to pheromone	5.91E-05	0.049	9	100
HD 12 - 18 m					
GO:0042391	Regulation of membrane potential	1.87E-07	0.001	32	105
GO:0055082	Cellular chemical homeostasis	2.69E-06	0.0081	50	221
GO:0048167	Regulation of synaptic plasticity	1.19E-05	0.0371	14	34
GO:0019226	Transmission of nerve impulse	1.37E-05	0.043	15	39
GO:0007155	Cell adhesion	1.77E-05	0.055	87	483

The probesets and genes that were significant over time are given in Table S1 and the full list of pathways in Table S2. Count is the total number of significantly differentially expressed probesets in the GO category and Global is the number of genes in that GO category.

### Effects of genotype on gene expression

Analysis between genotypes shows that 2557 probesets (1821 genes) are dysregulated between YAC128 and WT animals when using data from all time points (nominal *p* < 0.05), of which 1151 are up-regulated and 1106 are down-regulated in the YAC128 striata. Using an FDR threshold of *p* > 0.05, 87 probesets are dysregulated (Table 2), 53 down- and 34 up-regulated. Analysing the time points individually, at 6 months 1287 (856 genes), at 12 months 885 (482 genes) and at 18 months 2484 probesets (1514 genes) are altered in expression (nominal *p* < 0.05; Additional file 3: Table S3). Between 6 and 12 months 148 probesets (118 genes) are altered in abundance at both times (11.5 % and 6.0 % respectively; *p* = 0.02) and between 12 and 18 months there are 223 such probesets (164 genes, 25.2 % and 9.0 % respectively; *p* = 0.039) (Additional file 3: Table S3).

**Table 2:** Probesets dysregulated between genotypes

Gene ID	All			6 m			12 m			18 m		
	*p*-value	log2FC	AbsFC	*p*-value	log2FC	AbsFC	*p*-value	log2FC	AbsFC	*p*-value	log2FC	AbsFC
Down in YAC128												
Phex	1.13E-05	−0.645	1.564	1.58E-05	−0.649	1.568	2.09E-01	−0.596	1.512	2.84E-02	−0.691	1.615
Gm501	5.08E-05	−0.494	1.408	7.75E-04	−0.374	1.296	8.17E-02	−0.575	1.490	3.91E-02	−0.532	1.446
Plk5	5.08E-05	−0.448	1.364	6.26E-04	−0.358	1.282	2.27E-01	−0.409	1.328	1.29E-02	−0.577	1.492
Galnt13	1.76E-04	−0.442	1.359	3.52E-04	−0.437	1.353	6.91E-01	−0.374	1.296	8.24E-02	−0.516	1.430
Actn2	1.96E-04	−0.765	1.699	1.60E-04	−0.760	1.694	9.21E-01	−0.591	1.506	3.76E-02	−0.944	1.924
Dgat2l6	1.96E-04	−0.311	1.240	2.30E-04	−0.290	1.223	1.00E + 00	−0.230	1.173	1.42E-02	−0.412	1.331
Sec14l3	2.30E-04	−0.413	1.332	2.84E-03	−0.306	1.236	2.09E-01	−0.444	1.360	4.76E-02	−0.490	1.405
Odf4	2.67E-04	−0.384	1.305	8.07E-04	−0.384	1.305	6.37E-01	−0.391	1.311	2.33E-01	−0.377	1.299
Ptprv	9.42E-04	−0.322	1.250	7.77E-03	−0.255	1.193	7.66E-01	−0.314	1.243	1.08E-01	−0.396	1.316
Ddit4l	1.53E-03	−0.407	1.326	1.71E-04	−0.432	1.349	3.78E-01	−0.430	1.348	2.57E-01	−0.359	1.282
Ryr1	1.80E-03	−0.577	1.492	7.51E-04	−0.507	1.421	8.22E-01	−0.464	1.379	2.73E-02	−0.760	1.694
Npl	2.05E-03	−0.277	1.212	4.42E-02	−0.166	1.122	4.02E-01	−0.319	1.247	1.14E-01	−0.348	1.273
Cdc2l6	2.16E-03	−0.278	1.213	3.58E-04	−0.312	1.241	9.98E-01	−0.262	1.199	2.96E-01	−0.260	1.198
Krt9	2.72E-03	−0.393	1.313	2.47E-02	−0.289	1.221	8.22E-01	−0.423	1.341	2.06E-01	−0.468	1.383
Hvcn1	2.72E-03	−0.229	1.172	1.49E-03	−0.232	1.174	1.00E + 00	−0.151	1.110	9.09E-02	−0.305	1.235
Aoah	4.47E-03	−0.197	1.146	1.33E-02	−0.158	1.116	1.00E + 00	−0.210	1.157	2.26E-01	−0.223	1.167
Bmp2	4.57E-03	−0.286	1.219	4.42E-03	−0.263	1.200	3.78E-01	−0.354	1.278	4.72E-01	−0.240	1.181
Clspn	5.34E-03	−0.355	1.279	7.12E-03	−0.271	1.207	6.68E-01	−0.360	1.284	9.09E-02	−0.435	1.352
Kcnk13	8.57E-03	−0.164	1.120	1.79E-02	−0.135	1.098	7.97E-01	−0.203	1.151	4.72E-01	−0.152	1.111
Glul (2 probes)	9.47E-03	−0.170	1.125	7.37E-03	−0.147	1.107	3.79E-01	−0.222	1.166	4.83E-01	−0.142	1.103
Pkp2	1.02E-02	−0.282	1.216	9.44E-04	−0.313	1.242	3.78E-01	−0.370	1.292	7.26E-01	−0.164	1.120
Oprk1	1.48E-02	−0.456	1.372	1.49E-02	−0.307	1.237	1.00E + 00	−0.333	1.260	1.29E-02	−0.728	1.656
Cnr1	1.48E-02	−0.452	1.368	1.49E-02	−0.369	1.291	1.00E + 00	−0.442	1.359	2.09E-01	−0.545	1.459
Gpr155	1.48E-02	−0.329	1.256	6.12E-04	−0.438	1.355	1.00E + 00	−0.243	1.184	5.06E-01	−0.305	1.235
Zfp180	1.55E-02	−0.216	1.161	9.49E-04	−0.256	1.194	1.00E + 00	−0.165	1.121	3.30E-01	−0.226	1.170
Vrk1	1.93E-02	−0.243	1.184	1.58E-02	−0.223	1.167	1.00E + 00	−0.189	1.140	2.49E-01	−0.317	1.245
Farp2	2.05E-02	−0.154	1.112	4.16E-02	−0.123	1.089	1.00E + 00	−0.133	1.097	2.70E-01	−0.204	1.152
Il33	2.10E-02	−0.398	1.318	3.32E-04	−0.358	1.282	8.17E-02	−0.471	1.386	1.38E-01	−0.365	1.288
Tspan2	2.17E-02	−0.327	1.255	1.07E-02	−0.277	1.212	6.81E-01	−0.356	1.280	2.81E-01	−0.349	1.274
Scn4b	2.20E-02	−0.537	1.451	1.81E-03	−0.628	1.545	1.00E + 00	−0.401	1.320	3.58E-01	−0.582	1.497
Sbsn	2.20E-02	−0.183	1.135	1.56E-02	−0.175	1.129	1.00E + 00	−0.150	1.109	3.30E-01	−0.223	1.168
Ube2cbp	2.20E-02	−0.156	1.114	1.02E-02	−0.159	1.116	1.00E + 00	−0.160	1.117	5.38E-01	−0.150	1.110
Ppp1r9b	2.20E-02	−0.148	1.108	9.75E-03	−0.145	1.106	1.00E + 00	−0.080	1.057	1.34E-01	−0.220	1.165
Fn3k	2.20E-02	−0.143	1.104	2.64E-02	−0.121	1.088	7.91E-01	−0.178	1.131	5.71E-01	−0.129	1.094
Gpx6	2.24E-02	−0.652	1.571	2.59E-01	−0.243	1.183	1.00E + 00	−0.519	1.433	2.73E-02	−1.195	2.289
Erbb2ip	2.24E-02	−0.205	1.153	5.15E-03	−0.209	1.156	9.68E-01	−0.236	1.178	5.71E-01	−0.172	1.126
Lrrk2	3.02E-02	−0.372	1.294	4.21E-03	−0.412	1.331	1.00E + 00	−0.273	1.208	3.39E-01	−0.430	1.347
Ppp1r9a	3.21E-02	−0.265	1.201	1.15E-02	−0.269	1.205	1.00E + 00	−0.187	1.139	2.96E-01	−0.338	1.264
Traip	3.82E-02	−0.256	1.195	7.04E-02	−0.171	1.125	1.00E + 00	−0.146	1.106	6.65E-02	−0.453	1.369
Slc1a2	4.13E-02	−0.141	1.103	3.27E-02	−0.126	1.091	1.00E + 00	−0.164	1.120	6.16E-01	−0.133	1.097
Bank1	4.21E-02	−0.240	1.181	2.04E-03	−0.291	1.223	1.00E + 00	−0.131	1.095	2.73E-01	−0.298	1.229
Rnf8	4.72E-02	−0.116	1.084	7.03E-03	−0.130	1.094	1.00E + 00	−0.070	1.050	3.30E-01	−0.147	1.107
Up in YAC 128												
Tmc3	1.30E-04	0.480	1.395	1.49E-04	0.374	1.296	2.27E-01	0.395	1.315	6.46E-04	0.672	1.593
Polr2a	2.30E-04	0.288	1.221	2.54E-03	0.230	1.173	6.81E-01	0.279	1.214	6.12E-02	0.354	1.278
Il17rb	9.51E-04	0.238	1.179	2.36E-05	0.270	1.206	9.04E-01	0.198	1.147	9.09E-02	0.246	1.186
Fat1	1.27E-03	0.277	1.212	6.20E-04	0.290	1.223	1.00E + 00	0.276	1.211	2.70E-01	0.265	1.201
Ppia (6 probes)	2.16E-03	0.188	1.139	1.65E-03	0.189	1.140	1.00E + 00	0.151	1.110	1.45E-01	0.224	1.168
Lrrn3	2.16E-03	0.280	1.214	7.46E-04	0.310	1.240	9.98E-01	0.290	1.223	4.51E-01	0.240	1.181
Chdh	4.11E-03	0.181	1.134	9.58E-03	0.160	1.117	1.00E + 00	0.177	1.130	2.60E-01	0.207	1.154
Acy3	4.57E-03	0.292	1.224	2.42E-02	0.171	1.126	2.57E-01	0.322	1.250	4.05E-02	0.383	1.304
Stat1	7.72E-03	0.203	1.151	4.04E-02	0.140	1.102	1.00E + 00	0.227	1.170	2.26E-01	0.244	1.184
Smoc1	1.61E-02	0.265	1.202	3.00E-02	0.190	1.141	1.00E + 00	0.257	1.195	1.38E-01	0.348	1.273
Rnf122	2.05E-02	0.186	1.138	9.00E-02	0.112	1.081	1.00E + 00	0.181	1.134	1.45E-01	0.264	1.201
Cited2	2.05E-02	0.246	1.186	1.89E-02	0.225	1.169	1.00E + 00	0.225	1.169	3.64E-01	0.288	1.221
Enpp6	2.05E-02	0.365	1.288	2.66E-02	0.257	1.195	1.00E + 00	0.335	1.261	9.29E-02	0.504	1.418
Spata5	2.29E-02	0.177	1.131	4.76E-03	0.202	1.150	1.00E + 00	0.183	1.135	6.69E-01	0.146	1.107
Htr2a	2.58E-02	0.362	1.285	4.80E-02	0.284	1.218	7.53E-01	0.469	1.384	6.08E-01	0.334	1.260
Arsb (2 probes)	2.61E-02	0.176	1.130	1.13E-02	0.180	1.133	1.00E + 00	0.104	1.075	2.33E-01	0.244	1.185
Grhpr	3.21E-02	0.188	1.139	4.07E-02	0.144	1.105	1.00E + 00	0.152	1.111	1.65E-01	0.270	1.205
Zfp488	3.55E-02	0.236	1.178	5.66E-01	0.040	1.028	2.27E-01	0.331	1.257	7.49E-02	0.338	1.264
Pla2g4a	3.55E-02	0.253	1.191	3.29E-02	0.172	1.127	1.00E + 00	0.251	1.190	1.17E-01	0.335	1.261
Pou6f2	4.02E-02	0.170	1.125	5.31E-02	0.141	1.103	1.00E + 00	0.152	1.111	3.87E-01	0.218	1.163
Eya1	4.21E-02	0.246	1.186	2.01E-02	0.223	1.167	1.00E + 00	0.233	1.175	3.90E-01	0.281	1.215
Ifit1	4.21E-02	0.383	1.304	8.37E-03	0.380	1.301	1.00E + 00	0.388	1.308	4.67E-01	0.382	1.303
Nfe2l3	4.27E-02	0.169	1.125	4.26E-02	0.109	1.079	1.00E + 00	0.067	1.048	1.29E-02	0.332	1.258
Plekhh2	4.72E-02	0.200	1.148	1.95E-05	0.284	1.218	1.00E + 00	0.144	1.105	3.90E-01	0.171	1.126
Mobkl2b	4.82E-02	0.225	1.169	2.45E-02	0.171	1.126	1.00E + 00	0.254	1.192	2.73E-01	0.251	1.190

Probsets annotated to genes with a significant difference (FDR *p* < 0.05) between the two genotypes are given. Each gene corresponds to a single probeset unless indicated otherwise, the probeset of lowest p-value for all ages has been quoted in such cases. FC = fold change, AbsFC = absolute fold change.

**Fig. 1:**
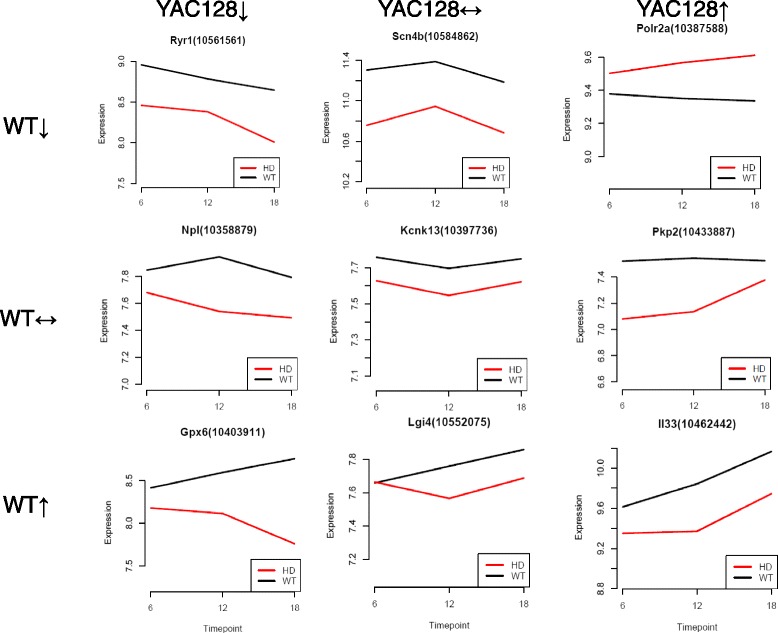
Patterns of expression identified using TANOVA. Each different pattern is illustrated by one gene showing a significant change in expression over time and between genotypes. The gene ID and the Affymetrix probeset ID are given. Expression is given as Log-2 fold change and the time points are in months. WT = wild-type and HD = YAC128

The TANOVA analysis (Fig. 1, Table 3) highlights a set of 146 probesets (125 genes) dysregulated between genotypes but also includes the trajectory of change in expression over time. Seventy-six genes are common to the TANOVA and LIMMA FDR *p* < 0.05 analyses. The TANOVA results show that if the trajectory of gene expression with increased age is towards decreasing mRNA abundance, then these genes are much more likely to be decreased in expression in the striata of YAC128 mice compared with wild-type: 39 such differentially expressed genes decreased in expression in YAC128 mice compared with 4 increased in expression. The opposite is true for genes increased in expression with age in the YAC128 mouse striatum: these are more likely to be increased in expression in the YAC128 striata: 48 increased compared with 22 decreased mRNAs. The full TANOVA plots of gene expression over time are given in Additional file 4: Figure S1.

**Table 3:** Differentially expressed genes between WT and YAC128 striata identified using TANOVA

		WT			
		Up	NC	Down	Total
	Up	45 (13↓,31↑)	12(3↓,8↑)	16(6↓,9↑)	73(22↓,48↑)
YAC128	NC	10(7↓,2↑)	8(5↓,3↑)	7(4↓,3↑)	25(16↓,8↑)
	Down	17(10↓,2↑)	9(8↓,1↑)	22(21↓,1↑)	48(39↓,4↑)
	Total	72	29	45	146

Numbers of genes significant for each TANOVA pattern of change over time, given as number (number of genes down-regulated in YAC128↓,number of genes up-regulated in YAC128↑): some genes showed a mixed pattern of change and have not been included. NC = no change. For instance in the top left hand corner, 45 genes were seen to increase in expression with age in YAC128 and wild-type striata, and of those 13 were reduced in expression in WT compared with YAC128 expression and 31 were increased in expression compared with YAC128 expression.

**Fig. 2:**
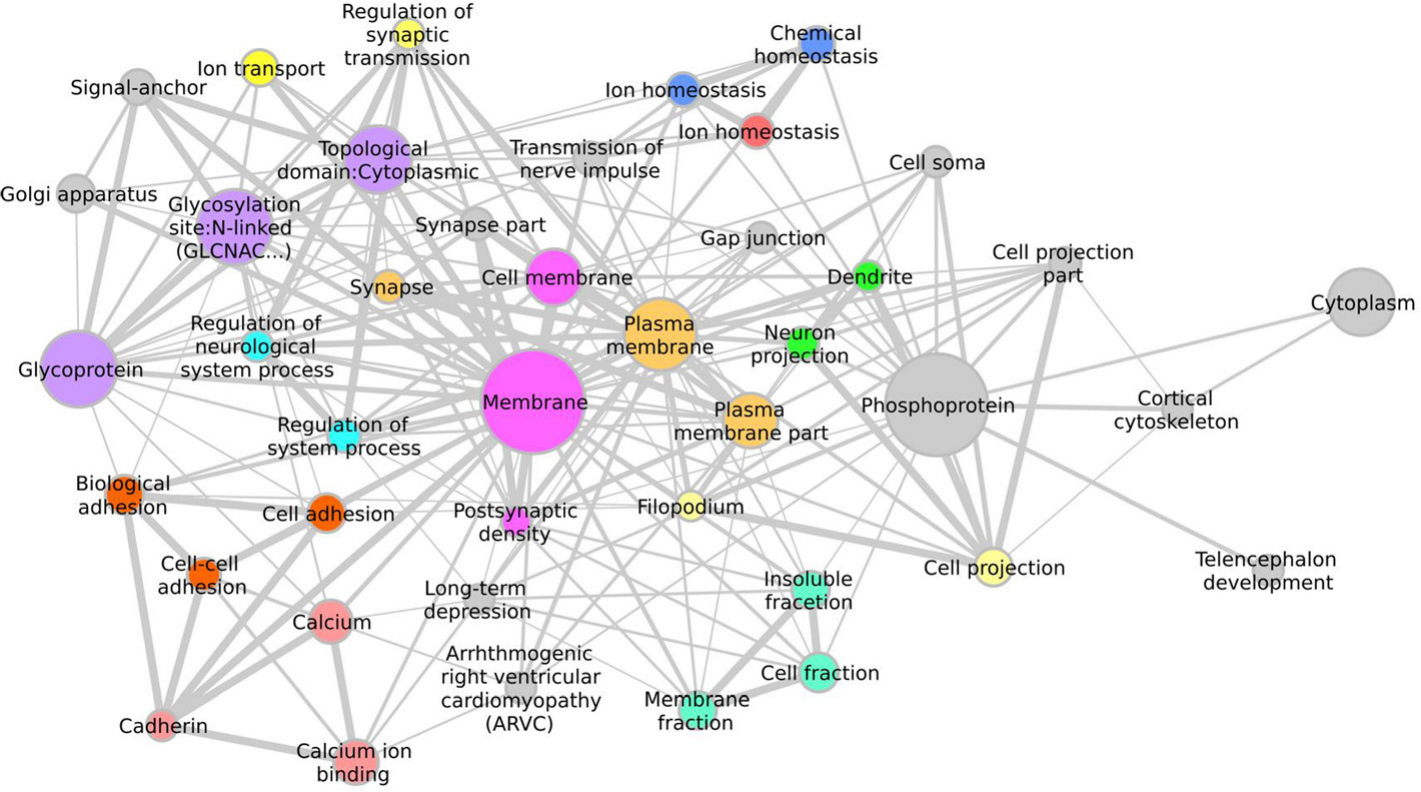
Pathways implicated by the gene expression differences in YAC128 mouse striata. List of differentially expressed genes were generated by t-test using a nominal *p* < 0.01; DAVID was used for the pathway analysis with a pathway filter *q* < 0.05. The pathway node size is proportional to gene membership and the edges joining nodes are weighted by gene overlap between nodes. Terms have been clustered into groups that contain 90 % genetic similarity on average. The most significant term of the cluster has been displayed. These terms have been further clustered at a level of 50 % average genetic similarity and colour-coded by cluster

An over-representation analysis using EASE and DAVID [31, 32] of the differentially expressed genes by TANOVA does not reveal any over-represented categories, probably due to the small numbers of genes within each pattern of change. However, an over-representation analysis of the full sets of genes identified by LIMMA analysis (Table 4) shows that the largest number of pathways are identified by the down-regulated genes and that these are mostly related to G-protein and other intracellular signalling pathways. An examination of the genes that contribute to these significant processes shows that there are substantial overlaps of genes amongst these significant categories and that the most specific pathway highlighted (the smallest) is GO:0019226, transmission of nerve impulse. Categories relating to nervous transmission and synaptic events appear as nominally significant even if they are not signficant once the FDR correction has been applied (Additional file 5: Table S4). In order to highlight the most significant functional relationships in the data we conducted a DAVID analysis [31, 32] visualised in cytoscape in Fig. 2. The most interconnected ontological term is membrane and all the other processes are related to membrane events: these include cell adhesion, neuronal projections, synaptic functions and transmission of nerve impulse.

**Table 4:** GO terms showing over-representation amongst genes differentially expressed in YAC128 compared with WT striata

ID	Term	FDR p	Count	Global
Genes down in YAC128				
GO:0007186	G-protein coupled receptor protein signaling pathway	2.65E-12	155	1616
GO:0007165	Signal transduction	9.18E-12	256	3253
GO:0003008	System process	1.05E-09	147	1624
GO:0007600	Sensory perception	1.45E-07	125	1364
GO:0007608	Sensory perception of smell	1.26E-06	104	1098
GO:0050801	Ion homeostasis	1.47E-04	34	239
GO:0050789	Regulation of biological process	2.26E-04	379	6038
GO:0055082	Cellular chemical homeostasis	2.35E-04	32	221
GO:0042391	Regulation of membrane potential	7.05E-04	20	105
GO:0007154	Cell communication	2.58E-02	24	218
GO:0019226	Transmission of nerve impulse	3.42E-02	10	39
Genes up in YAC128				
GO:0019884	Antigen processing and presentation of exogenous antigen	1.74E-02	8	26

The full list of differentially expressed genes between genotypes is given in Table S3 and the full pathway analysis in Table S4. Count is total number of significantly differentially expressed probesets in that GO category and Global is the total membership of that GO category.

### Comparison with human HD and other mouse models

**Fig. 3:**
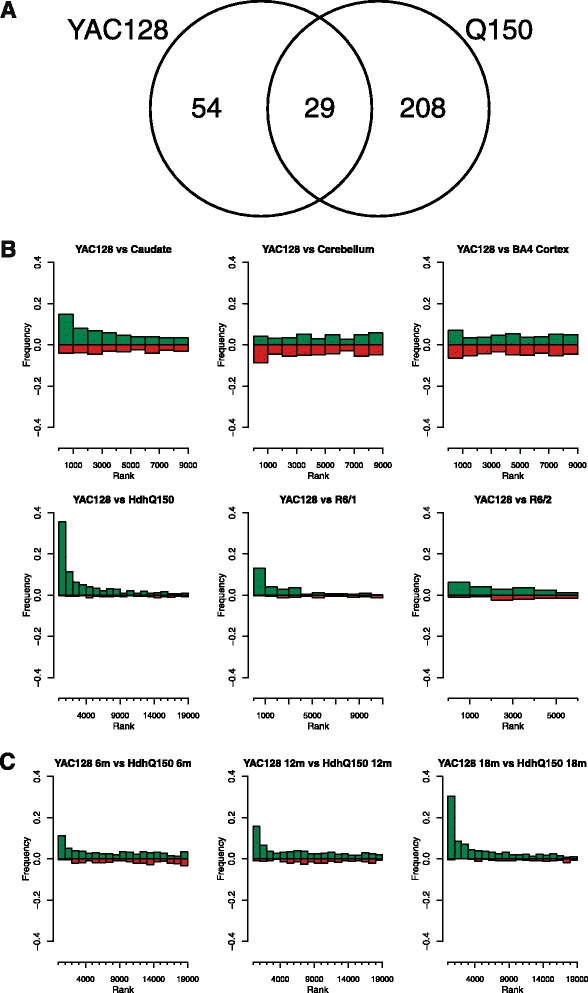
Correlation of direction of expression changes in YAC128 striata, other mouse models of HD and human HD caudate. **a** shows the overlap of genes within the FDR adjusteddifferentially expressed genes from the YAC128 compared with the HdhQ150 mouse striata. In B and C frequency  represents the fraction of the top 200 HdhQ150 expression changes that map to a particular bin of ranked data (1000 genes per bin) in the other dataset, which is then split to concordant or discordant direction of expression change. A higher frequency of concordant (green) rather than discordant (red) in the first bins indicates a similarity between the YAC128 caudate and other model or human HD gene expression signature. **b** shows the comparison of YAC128 striatal gene expression with human HD brain regions [18] and the comparison with other mouse models and **c** shows the comparison at the same time points with the HdhQ150 striatal gene expression.

A straightforward examination of the overlap between the YAC128 and HdhQ150 FDR controlled lists of differentially expressed genes shows that more genes are common to the two lists than might be expected (*p* <10^−4^; Fig. 3a). However this only examines a very small part of each genelist and the relationship of alterations in gene expression extends over a much more substantial proportion of the genes surveyed: in addition the simple analysis above does not take direction of change in gene expression into account. It is also hard to compare across species and gene expression platforms. To gain a more detailed understanding of the relationship between the striatal gene expression changes between the YAC128, human brain and other mouse model striata we examined the overlap by direction in ranked bins of the gene expression differences. The overlap between the top 1000 probesets altered in abundance between the YAC128 striata and human caudate is substantial (179/1000 probesets, *p* = 0.018, 80/674 orthologous genes (that are coding genes in both species)) [18]. There is also a significant overlap with human cerebellum (117/1000 probesets, *p* = 0.008, 47/674 orthologous genes), human BA4 cortex (124/1000 probesets, p =0.016, 55/674 orthologous genes) and BA9 cortex (71/1000 probesets, *p* = =0.025, 32/674 genes). The YAC128 striatal genes altered in abundance overlapped significantly with those seen in the HdhQ150 striata (272/1000 probesets, *p* <10^−4^, 132/779 genes) and also R6/1 (113/1000 probesets, *p* = 0.001, 103/779 genes) and R6/2 whole brain (65/1000 probesets, *p* = 0.012, 62/779 genes) [19, 33]. The direction of these changes is also largely concordant (Fig. 3b). Conducting the comparison at the individual time-points reveals that the substantial overlap between the expression profile of these tissues is already significant at 6 months and becomes increasingly significant over time (Additional file 6: Figure S2). A direct comparison of the YAC128 and HdhQ150 gene expression profiles at the time points for which gene expression data are available shows that concordance and overlap increase over time although they are already substantial by 6 months (Fig. 3c). To ensure that the overlaps observed were not just a result of non-specific striatal pathology, we compared the YAC128 gene expression data in this study to that from a study of MPTP effects on gene expression which also generated gene expression profiles in mouse striatal tissue [34]. We detected no significant overlap of genes differentially expressed between the two studies (19/1000 genes, NS).

Despite the substantial overlap in gene expression profiles, there are genes whose variation in expression differs between the two models. Differences between genotype and model were assessed using ANOVA. This was done by fitting a regression model containing the main effects of genotype and model, together with their interaction term. A significant (*p* < 0.05) interaction term was taken as evidence of a difference in gene expression between models. There were 869 probesets with a significant interaction term (*p* < 0.05) (Table 5, Additional file 7: Table S5). Examination of enrichment does not reveal any significant functional pathways identified by these genes, but the most significant individual gene is *Htt* itself.

**Table 5:** Genes that show significantly different patterns of differential expression between YAC128 and HdhQ150 striata

Symbol	Difference *p*	Q150 FC	YAC FC	Abs FC diff	Description
Actn2	1.07E-03	−0.19	−0.79	0.60	Actinin alpha 2
Htr2a	2.12E-04	−0.15	0.37	0.52	5-hydroxytryptamine (serotonin) receptor 2A
Ifit1	5.88E-03	−0.03	0.41	0.44	Interferon-induced protein with tetratricopeptide repeats 1
Galnt13	5.89E-05	−0.03	−0.45	0.42	UDP-N-acetyl-alpha-D-galactosamine:polypeptide N-acetylgalactosaminyltransferase 13
Htt	3.19E-10	−0.32	0.09	0.41	Huntingtin
Lgals3bp	9.45E-03	−0.04	0.36	0.40	Lectin, galactoside-binding, soluble, 3 binding protein
Iqub	3.74E-03	−0.33	0.04	0.37	IQ motif and ubiquitin domain containing
Ifi27l1	3.33E-04	−0.06	0.31	0.37	Interferon, alpha-inducible protein 27 like 1
Usp18	1.32E-03	−0.08	0.28	0.36	Ubiquitin specific peptidase 18
Dpp10	2.03E-03	−0.07	0.26	0.34	Dipeptidylpeptidase 10
Pkp2	9.91E-05	0.04	−0.29	0.33	Plakophilin 2
Olfr174	4.74E-03	0.15	−0.18	0.33	Olfactory receptor 174
Trim30	5.90E-04	−0.11	0.21	0.32	Tripartite motif-containing 30
Trpc6	6.67E-03	−0.27	0.02	0.29	Transient receptor potential cation channel, subfamily C, member 6
Zfp185	8.12E-03	−0.09	0.19	0.28	Zinc finger protein 185
Grk4	4.41E-03	−0.06	0.21	0.27	G protein-coupled receptor kinase 4
Acot9	4.17E-04	−0.10	0.17	0.26	Acyl-CoA thioesterase 9
Lgals2	6.58E-03	0.29	0.05	0.25	Lectin, galactose-binding, soluble 2
Rreb1	2.71E-03	−0.11	0.14	0.24	Ras responsive element binding protein 1
Dpysl5	1.39E-03	0.20	−0.04	0.24	Dihydropyrimidinase-like 5
Ceacam2	9.51E-03	−0.16	0.08	0.24	Carcinoembryonic antigen-related cell adhesion molecule 2
Wdr78	5.63E-03	−0.27	−0.03	0.24	WD repeat domain 78
Dnajc5g	5.58E-03	−0.11	0.13	0.23	DnaJ (Hsp40) homolog, subfamily C, member 5 gamma
Ccdc108	8.86E-03	−0.06	0.17	0.23	Coiled-coil domain containing 108
Plac8l1	3.01E-03	0.13	−0.10	0.23	PLAC8-like 1
Rell2	9.35E-03	0.19	−0.04	0.22	RELT-like 2
Tnfsf8	1.67E-03	0.10	−0.12	0.22	Tumor necrosis factor (ligand) superfamily, member 8
Pabpc1l2b	7.47E-03	0.20	−0.01	0.22	Poly(A) binding protein, cytoplasmic 1-like 2B
Slc13a5	7.14E-03	−0.15	0.07	0.21	Solute carrier family 13 (sodium-dependent citrate transporter), member 5
Irf9	1.68E-03	−0.05	0.16	0.21	Interferon regulatory factor 9
Ranbp2	5.60E-03	−0.17	0.04	0.21	RAN binding protein 2
Fut4	7.54E-03	−0.16	0.05	0.21	Fucosyltransferase 4
Tpbg	4.70E-03	0.09	−0.11	0.21	Trophoblast glycoprotein
Rnf213	4.48E-03	−0.01	0.20	0.20	Ring finger protein 213
Speer2	4.22E-03	0.14	−0.06	0.20	Spermatogenesis associated glutamate (E)-rich protein 2
V1rb3	5.18E-03	0.08	−0.12	0.20	Vomeronasal 1 receptor, B3
Setd1b	5.82E-03	0.13	−0.07	0.20	SET domain containing 1B
Dtl	1.26E-03	−0.10	0.09	0.20	Denticleless homolog (Drosophila)

Genes with a significant interaction between YAC128 and HdhQ150 differential gene expression where the absolute fold change difference is > 0.20. FC = fold change, Abs FC = absolute fold change.

### Comparison of inclusion prevalence between mouse models

**Fig. 4:**
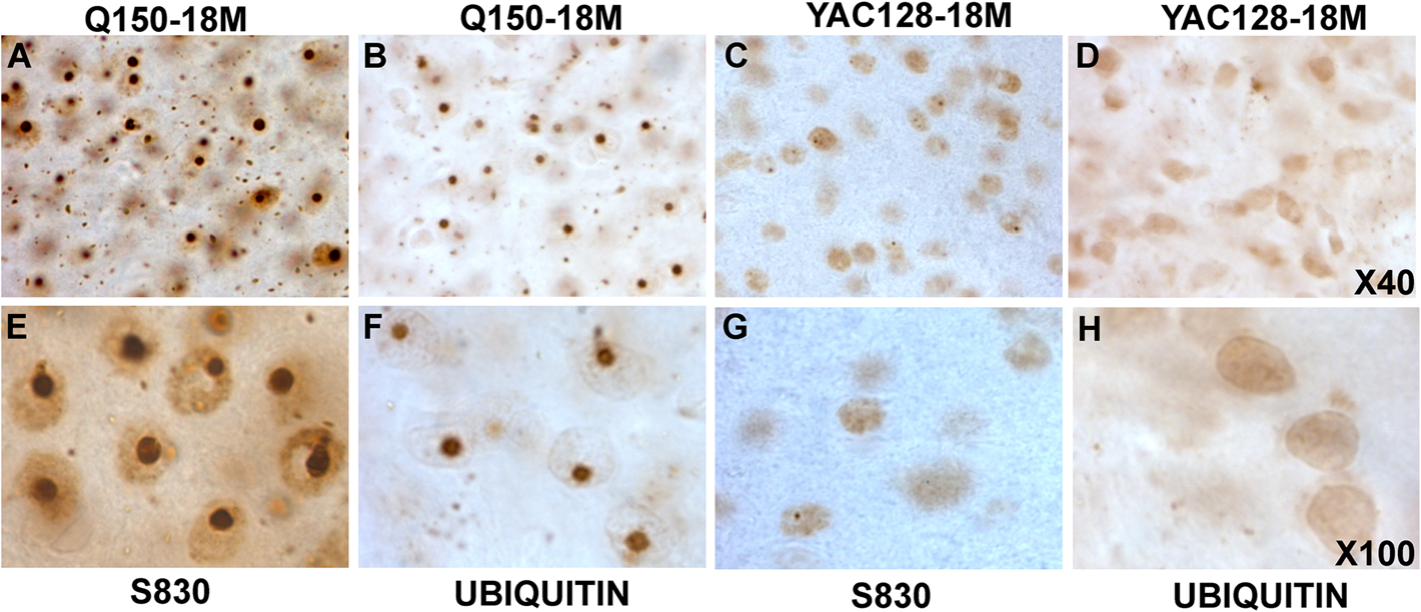
Comparative mutant HTT pathology in the HdhQ150 (panels **a**, **b**, **e**, **f**) and YAC128 (panels **c**, **d**, **g**, **h**)  mouse brain. Comparative immunohistochemistry in brains from 18 month old YAC128 or HdhQ150 mice using either the mutant HTT specific antibody S830 or the anti-ubiquitin at 100x magnification

To assess whether the gene expression changes correlated with HTT-specific inclusions in the YAC128 and HdhQ150 mouse models we examined mutant HTT and ubiquitin distribution in mouse brain using immunohistochemistry (Fig. 4). All regions of the HdhQ150 mouse brain showed widespread S830 mutant HTT positive inclusions (Fig. 4a). In contrast the YAC128 mouse brain at 18 months showed few frank inclusions (Fig. 4c), though there is nuclear filling in all regions and a few small inclusions in the hippocampus. The HTT in the Q150 mice is entirely mouse and all mutant, whereas YAC128s have human mutant HTT and endogenous mouse HTT. In order to ascertain that the S830 was detecting similar inclusions we also assessed inclusion load using anti-ubiquitin (Fig. 4b and d). The anti-ubiquitin antibody also detects the inclusions in the Q150 mouse brain, though they are less frequent than the S830 positive inclusions, showing that the inclusions shown by the HdhQ150 brain are not a direct result of the relevant mutant HTT epitope only being available to the S830 antibody in the HdhQ150 but not the YAC128 brains.

## Discussion

The data demonstrate that the gene expression changes in the YAC128 mouse striata are similar to those in the HdhQ150 striata at all ages [35]. These results are directly comparable as samples from mice of the same age were arrayed on the same chip. The similarity increases with age which is most likely to indicate a convergent gene expression phenotype in the striata of these models as the effects of the mutation become more marked and overcome differences between the models. It also demonstrates that the trajectory of molecular changes in the striata of these two differently constructed model lines parallel each other very well, despite differences apparent in the onset of their phenotypic changes [27, 28, 36].

The similarities in RNA changes are also paralleled in the human HD brain, especially in the caudate [18]. It is unsurprising that the human caudate gene expression profile should more closely parallel that of the mouse striatum than those of the human cortex or cerebellum, as the mouse caudate is contained within the striatal tissue analysed. These data therefore indicate that the gene expression profiles of these two mouse models of HD are convergent over time, and this is reinforced by comparisons with other models and with human caudate.

One major difference between these two models of HD is in the development of HTT-positive inclusions [30, 37]. The YAC128 model develops frank nuclear inclusions in the brain relatively late, from 15 months of age onwards, whereas the HdhQ150 model has such inclusions present from 5 months of age. The reason for this difference is not understood, though it is possible that the human and mouse proteins show differences in reactivity to the S830 antibody: however, the ubiquitin immunohistochemistry also shows that intranuclear inclusions are present in the HdhQ150 but not the YAC128 mouse brain at 5 months. Fewer ubiquitin-positive inclusions are observed than S830 positive inclusions which is consistent with previous data indicating that ubiquitin positive staining is a later event than mutant-HTT positive staining of inclusions in mouse brain [38]. However, the trajectory of nuclear filling with mHTT immunoreactivity followed by inclusion formation is common to both models and thus it seems most likely that this is a true difference in mHTT aggregation in these models. The concentrations of mHTT present in the YAC128 brain are probably similar to that in the HdhQ150 model as we noted reduced expression of mHTT in the HdhQ150 model such that it probably only expresses around half the WT endogenous level in brain [13, 39]. However, the presence of normal mouse HTT might inhibit inclusion formation. If endogenous mouse HTT is recruited into the inclusions then the presence of heterozygous protein differences is known to slow aggregation of the cognate proteins in other neurodegenerative diseases where protein inclusions are present in the disease, for instance in prion-related disease [40, 41]. While we have noted there are strong similarities in the striatal gene expression changes between these models, there are also differences, although the differences do not highlight any specific functional pathways: nevertheless these differences might contribute to the difference in inclusions observed. Discordant inclusion formation and pathogenic effects have been noted previously in a number of different systems [42, 43] though the connection with similar gene expression profiles in the face of differential inclusion formation has not been made previously.

The significant differences between the gene expression profiles of the striata from the YAC128 and HdhQ150 lines do not highlight any obvious functional differences between the two models that could account for the observed differences in inclusion prevalence. *Grk4* is close to the *Htt* locus in mice but it is not contained within the YAC construct used to generate these mice [13]: thus this cannot explain the increased expression of this gene. Other than this the significantly altered genes are not in the same chromosomal locations, so no chromosomally specific effect can be inferred that is due to the direct action of the transgene. The gene most significantly altered in expression is *Htt* itself and this is expected in light of the substantial down-regulation of mHTT in the HdhQ150 homozygous knock in striata [39].

Most of the genes that are differentially expressed between the models show increased expression in YAC128 striata and it is possible that this is due to the increased expression of HTT itself over the endogenous HTT expression. Mutant HTT can rescue the lethal effects of knocking out WT HTT [4, 44] so the mutant protein may well be exerting functional effects in relation to its normal as well as its pathological function. The expression of the YAC transgene in the YAC128 animals is close to the level of expression of the endogenous mouse gene [45]. The differential changes could potentially be related to the normal function of HTT through the effects of increased overall huntingtin expression and thus enhancement of the normal function. The gain in weight of the YAC128 animals over time compared with most HD mouse models has been suggested to be the result of the third copy of *HTT* and higher huntingtin expression [46]. There is, however, no overlap with the genes found to be altered in *Htt* null cell lines [47], although this could be the result of the very different biological systems studied and the different chips used.

*Htr2a* is expressed more highly in the YAC128 than the HdhQ150 striata. HTR2A in humans is the major serotonin receptor in the brain and is a target of the SSRI citalopram, which downregulates its expression [48–50]. The potentially depressive-like symptoms noted in the YAC128 animals [26] might therefore relate to alterations in the expression of this receptor. The deficits in the forced swim test in the YAC128 mice are thought to be a surrogate test for a syndrome related to depression in mice and this alteration in gene expression might underlie this observation. The SSRI fluoxetine did not improve performance in the forced swim task in YAC128 mice, but this test is confounded in these mice by the motor deficit and it is not clear that fluoxetine has the same effects on Htr2a as citalopram. In addition, the improvement in symptoms seen in mice treated with SSRIs is thought to be mediated by BDNF levels: it is possible that these are so compromised in the YAC128 mice that the drugs are incapable of improving them although levels of *Bdnf* RNA measured in the striata are unchanged in the cohort of animals that we used. However, given the prevalence of depressive symptoms in HD patients [26, 51–53] this may shed light on their mechanism.

The differences in gene expression with age in these animals is not the same as for the HdhQ150 animals. There are fewer changes and they do not highlight as many pathways, or the same pathways, as in the HdhQ150 mouse striata, especially between 6 and 12 months [35]. Between 12 and 18 months more pathways are highlighted some of which relate to perception of smell and neurological processes and notably genes for the perception of smell were enriched between 6 and 12 months in the HdhQ150 cohorts. It is not clear why these differences exist, and it must be remembered that these are not true longitudinal data (the same measurements in the same animals) but rather pseudo-longitudinal: the animals are maintained in the same way and are on the same genetic background but are different animals at the different time points. There may be subtle differences in the housing over time that have contributed to these differences in striatal gene expression profiles with age.

Examining the effects of genotype with age using TANOVA shows that genes that are down-regulated in the WT animal striata with age are also down-regulated in the Q150 knock in homozygote striata. This may indicate that the processes underlying the down-regulation of gene expression in HD model striata are related to those in normal ageing. The genes highlight a number of differentially regulated pathways including those involved in G-protein mediated signal transduction and homeostasis, although there is no direct overlap with the enriched pathways seen in the Q150 striata, despite the strong concordant overlap in the expression of individual genes [39].

## Conclusions

The related pathways of cell adhesion, neuronal projections, synaptic functions and transmission of nerve impulse seen in the DAVID analysis for enriched pathways indicates that the maintainance and regulation of connections between neurons is central to the molecular pathogenesis in YAC128 striatum.

The similarities in striatal gene expression differences between this HD model and human caudate and with other mouse models indicate that similar molecular processes are probably occurring. These appear to occur despite differences in inclusion formation. This indicates that the presence of HTT-positive inclusions is not necessary for these changes to occur. This separation of inclusion formation from the molecular consequences of the *Htt* mutation is worthy of further investigation as many studies in *in vitro* systems use inclusion formation as a measurable end-point to assess the effects of potential therapeutics.

## Methods

### Samples

Heterozygous YAC128 mice on a C57BL6/J background [13] were bred in house and genotypes ascertained using tail tip DNA (Laragen Inc., Los Angeles). Mice were culled by cervical dislocation at the same point in the light phase of the diurnal cycle. CAG repeat lengths in the YAC128 animals were 121 and no variation from this was detected. WT and hemizygous YAC128 animals of both sexes were used in the experiments (57 % male). The animals were housed as sex matched littermate groups and had access to food and water *ad libitum*. All experiments were carried out in accordance with the United Kingdom Animals (Scientific Procedures) Act of 1986, and subject to local ethical review (Project licence PPL30/1968 and PPL30/2305). The behavioural data relating to the complete cohort of mice are given in Brooks et al. [27].

### Gene expression

From this experimental group, 15 hemizygous YAC128 (7 female and 8 male) and 14 WT (Hdh^+/+^) mice (6 female and 9 male) were used for gene expression studies. Brains from age matched mice from each genotype were harvested at 6, 12 and 18 months and micro-dissected into striatum, motor cortex, cerebellum, prefrontal cortex and hippocampus. The dissected brain samples were snap frozen in liquid nitrogen and stored at −80 °C.

Total RNA was extracted from micro-dissected striata for gene expression analysis as previously described [19, 35]. RNA quality was determined using an Agilent RNA 6000 Nano Kit and Agilent 2100 Bioanalyser (Agilent Technologies, Santa Clara, USA). Samples with RIN (RNA integrity number) values greater than 7.5 were selected for subsequent analysis. For each RNA sample, cDNA was generated from 100 ng total RNA using an Ambion® WT expression kit (Applied Biosystems Carlsbad, California, USA), followed by fragmentation, labelling and hybridisation to a Mouse GeneChip Gene 1.0 ST Array. An Affymetrix WT Terminal Labelling and Hybridisation kit was used according to the manufacturer’s protocol. Gene Chips were processed using a Fluidics station 450 and a GeneChip scanner 3000 7G (Affymetrix UK Ltd, High Wycombe UK).

### Statistical methods

#### Gene expression analysis

An analysis of GeneChip expression data was undertaken using R/Bioconductor. Expression values were computed using robust multichip average (RMA) (affy package [54]), with testing for differential gene expression by age or genotype performed using moderated t-tests in LIMMA [55]. Changes in gene response over time were identified using TANOVA [56]. Genes with a false discovery rate (FDR) [57] corrected *p* < 0.05 were extracted and the data for these genes classified in three patterns representing an up, no change or down difference in expression over the time course (this was done separately for the WT and YAC128 animals tagging each gene with its highest correlation to theoretical expression profiles). The gene expression data are available through GEO accession number GSE70656.

#### Determining biological themes

The resultant gene lists from the differential gene expression, time course ANOVA and behaviour/expression correlation were analysed for over-representation of genes in pathways against GO Biological Process gene sets using the Bioconductor GOstats package with the conditional hypergeometic test (which only uses those terms that were not already significant when testing a higher order (parent) term). Changes in expression of genes in GO gene sets were assessed using Gene Set Analysis [58] against the whole dataset. The biological themes were further analysed using the DAVID database [31, 32] (with the appropriate background gene list selected for the make of microarray at the website). An input list was chosen for all probesets significantly differentially expressed at a nominal *p*-value < 0.01. The output from this and visualised on Cytoscape (version 2.8.3) [59, 60], via the Enrichment Map plugin.

#### Comparison with other gene expression data

Comparison with differentially expressed genes from human HD brain [18] and other HD models [19, 33] was calculated using hypergeometric tests on the top 1000 ranked genes in gene lists for differently expressed genes between WT and YAC128. To enable comparisons between different array platforms where the species was identical, probesets in gene lists were first converted to unique EntrezGene IDs and the overlap calculated using these. Where overlaps were made between data for different species, data was first converted to Entrez Gene IDs and then to Homologene IDs which were used to calculate the overlap between lists. In addition, a graphical representation of the overlap, along with information about the relative direction of changes was generated using the method of Kuhn [17]. Genes differentially expressed between YAC128 and HdhQ150 striata were identified by a significant interaction term in the ANOVA.

#### Histology

Tissue was processed and immunohistochemistry carried out as previously [61]. The ubiquitin antibody was Mouse anti-ubiquitin from Invitrogen used at a dilution of 1/1000 in TBS which stains huntingtin positive inclusions [62]. S830, which recognises mutant HTT was a kind gift from Gillian Bates [63].

## Additional files

Additional file 1: Table S1. Genes altered in expression with age common to both WT and YAC128 caudate. (XLS 75 kb) Additional file 2: Table S2. Over-representation analysis of the gene expression differences with age in YAC128 and WT striata (XLS 116 kb) Additional file 3: Table S3. Genes differentially expressed between WT and YAC128 striata. (XLS 393 kb) Additional file 4: Figure S1. TANOVA patterns of genes differentially expressed between WT and YAC128 caudate. Plots are sorted by pattern of changed expression and each of the 9 patterns is indicated at the end of the relevant section. The gene ID and the Affymetrix probeset ID are given. Expression is given as Log-2 fold change and the time points are in months. WT = wild-type and HD = YAC128. (PDF 65 kb) Additional file 5: Table S4. Over-representation analysis of the 2557 genes nominally significantly differentially expressed between YAC128 and WT striata (XLS 61 kb) Additional file 6: Figure S2. Analysis of the overlap of changes in gene expression in the YAC128 striatum at A. 6 months of age B. 12 months of age and C. 18 months of age compared with other HD model mouse striata and human brain. Frequency represents the fraction of the top 200 HdhQ150 expression changes that map to a particular bin of ranked data (1000 genes per bin) in the other dataset, which is then split to identify concordant or discordant direction of expression change. A higher frequency of concordant (green) rather than discordant (red) in the first bins indicates a similarity between the YAC128 caudate and other model or human HD gene expression signature. (PDF 59 kb) Additional file 7: Table S5. The 869 probesets that are differentially expressed between YAC128 and HdhQ150 striata. (XLS 137 kb)
