# Supplementary material for: Similar striatal gene expression profiles in the striatum of the YAC128 and HdhQ150 mouse models of Huntington’s disease are not reflected in mutant Huntingtin inclusion prevalence
Source: BMC Genomics. 2015 Dec 21;16:1079. doi: 10.1186/s12864-015-2251-4 (PMC4687121; doi:10.1186/s12864-015-2251-4)
Supplement: Additional file 4: Figure S1. — TANOVA patterns of genes differentially expressed between WT and YAC128 caudate. Plots are sorted by pattern of changed expression and each of the 9 patterns is indicated at the end of the relevant section. The gene ID and the Affymetrix probeset ID are given. Expression is given as Log-2 fold change and the time points are in months. WT = wild-type and HD = YAC128. (PDF 65 kb) [file 12864_2015_2251_MOESM4_ESM.pdf]

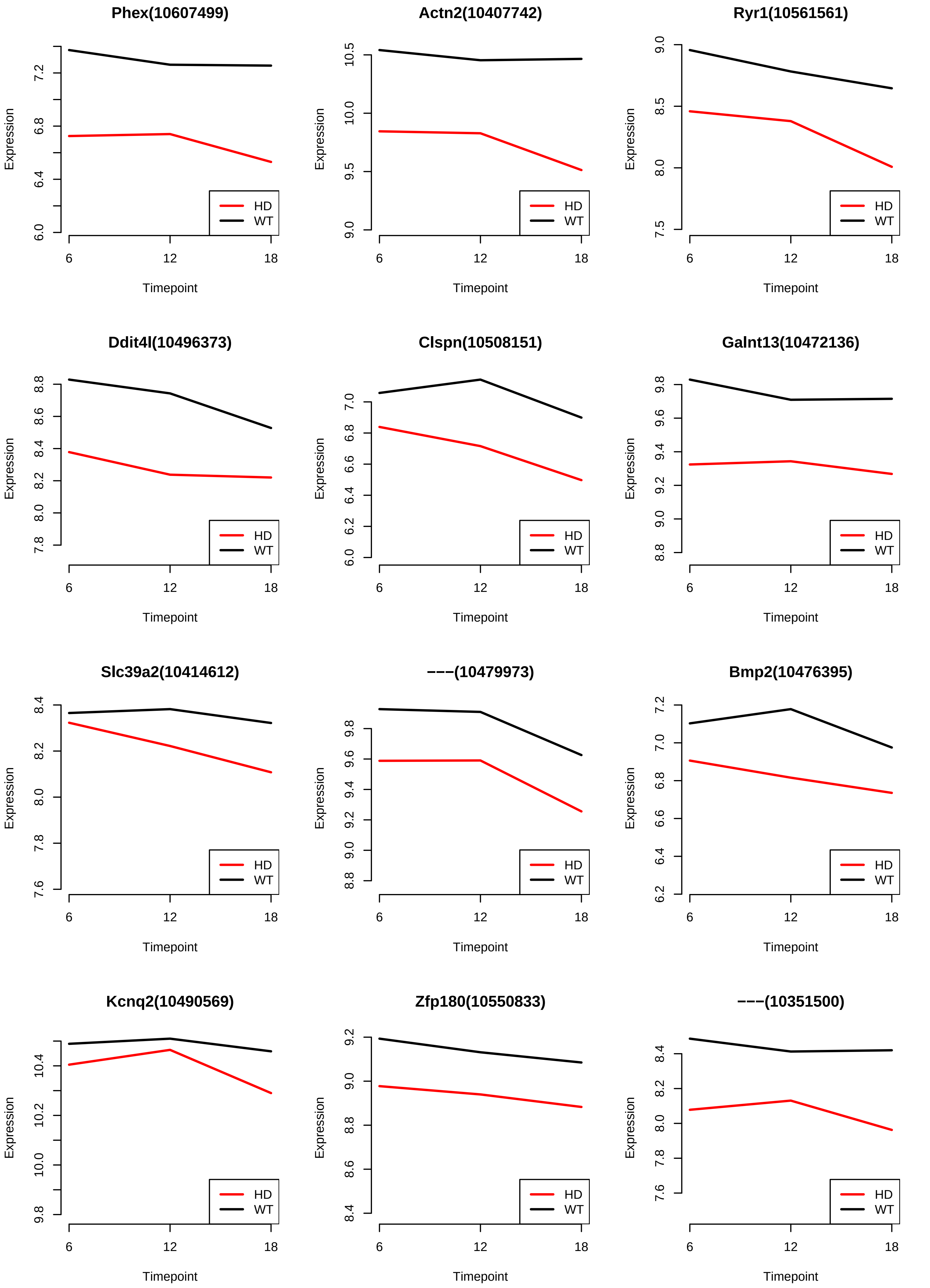
Phex(10607499)
Actn2(10407742)
Ryr1(10561561)
Ddit4l(10496373)
Clspn(10508151)
Galnt13(10472136)
Slc39a2(10414612)
---(10479973)
Bmp2(10476395)
Kcnq2(10490569)
Zfp180(10550833)
---(10351500)
Expression
Timepoint
6
12
18
HD
WT

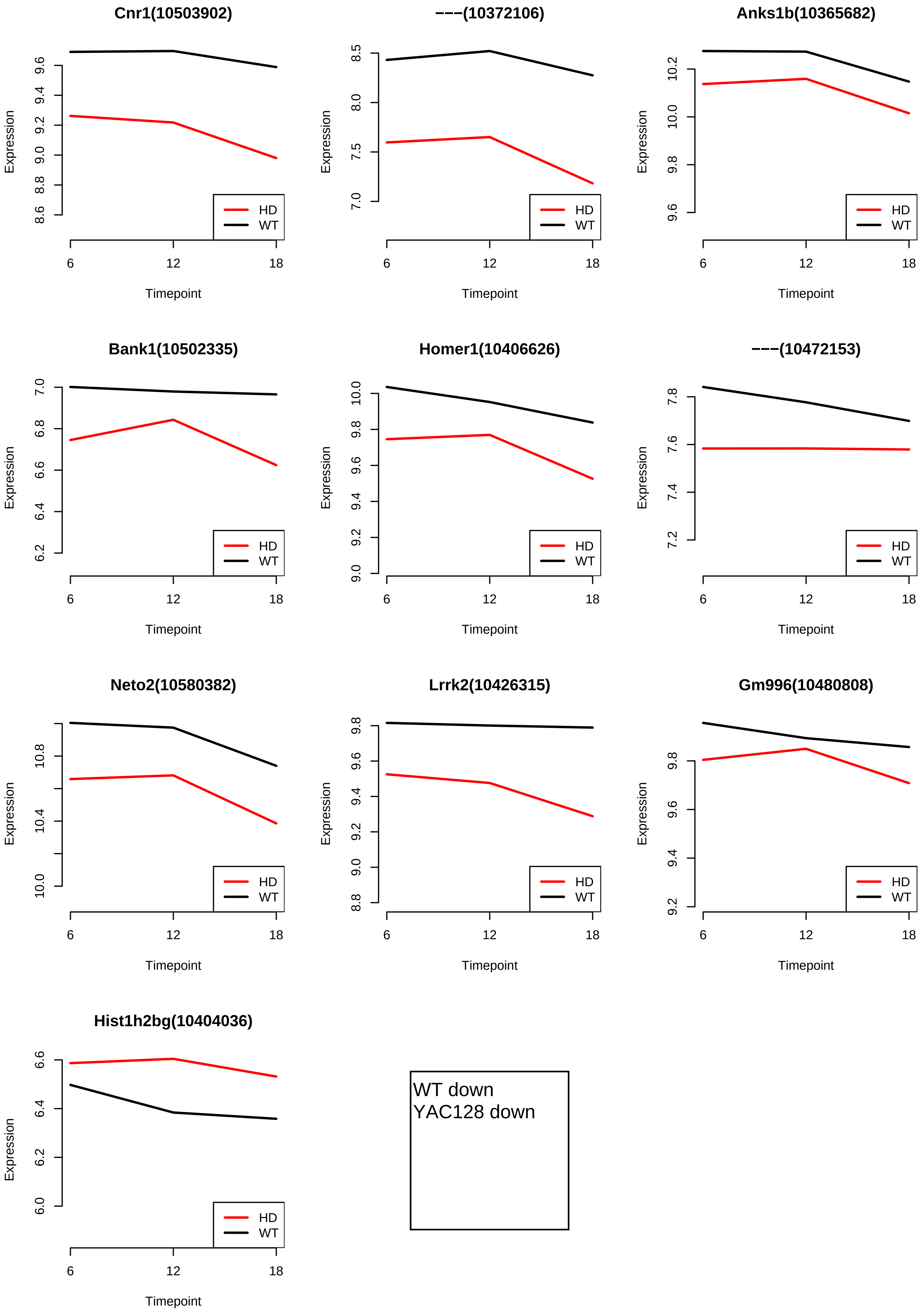
Cnr1(10503902)
---(10372106)
Anks1b(10365682)
Bank1(10502335)
Homer1(10406626)
---(10472153)
Neto2(10580382)
Lrrk2(10426315)
Gm996(10480808)
Hist1h2bg(10404036)
Expression
Timepoint
6
12
18
HD
WT
WT down
YAC128 down

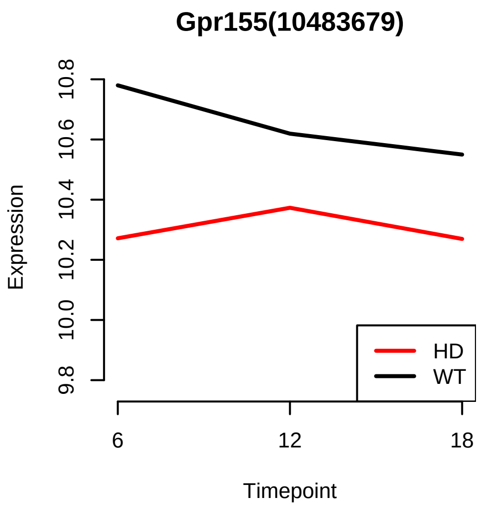

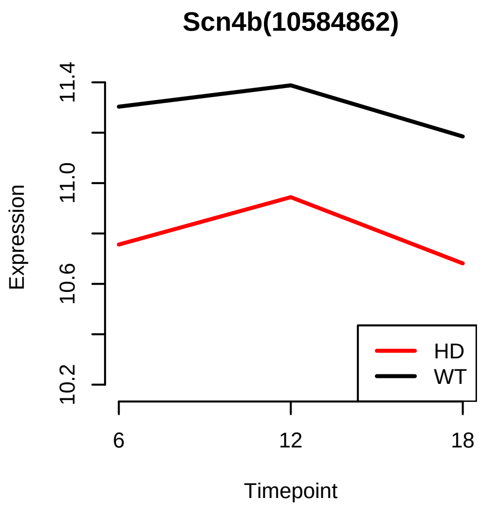

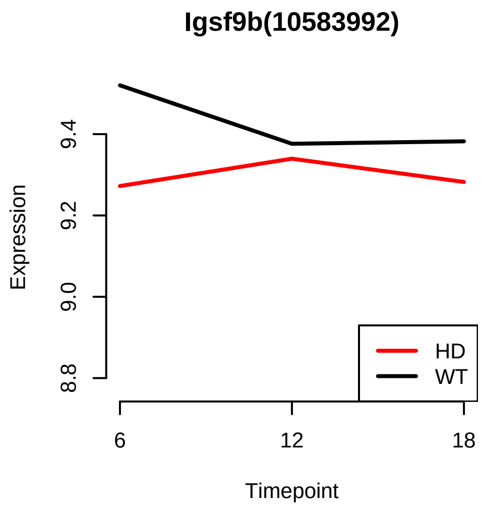

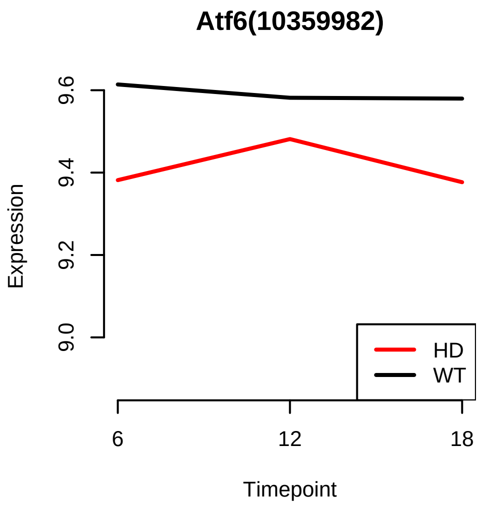

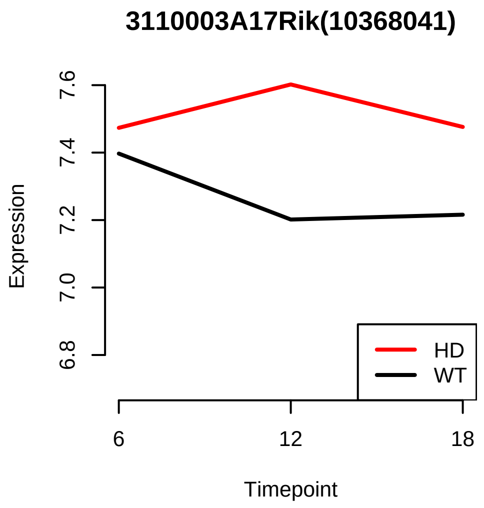

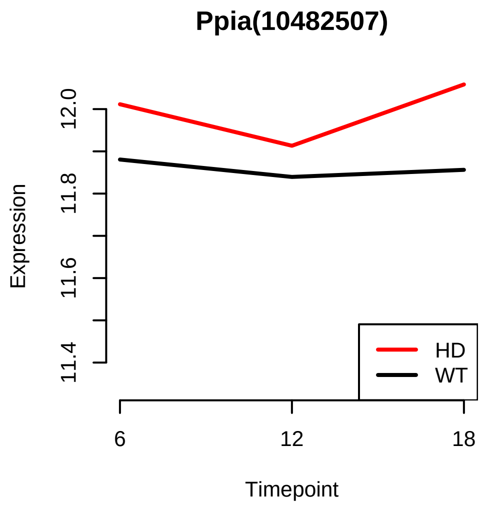

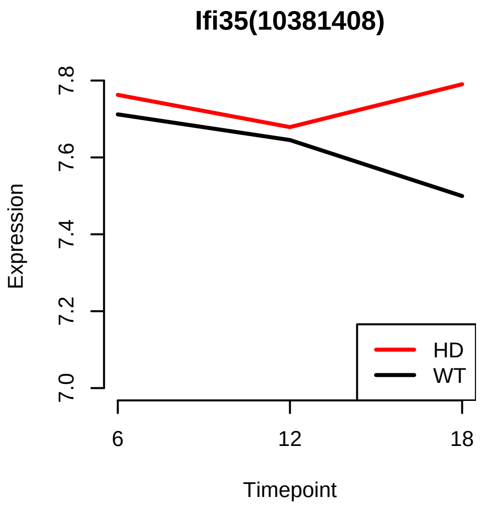

WT down
YAC128 no
change

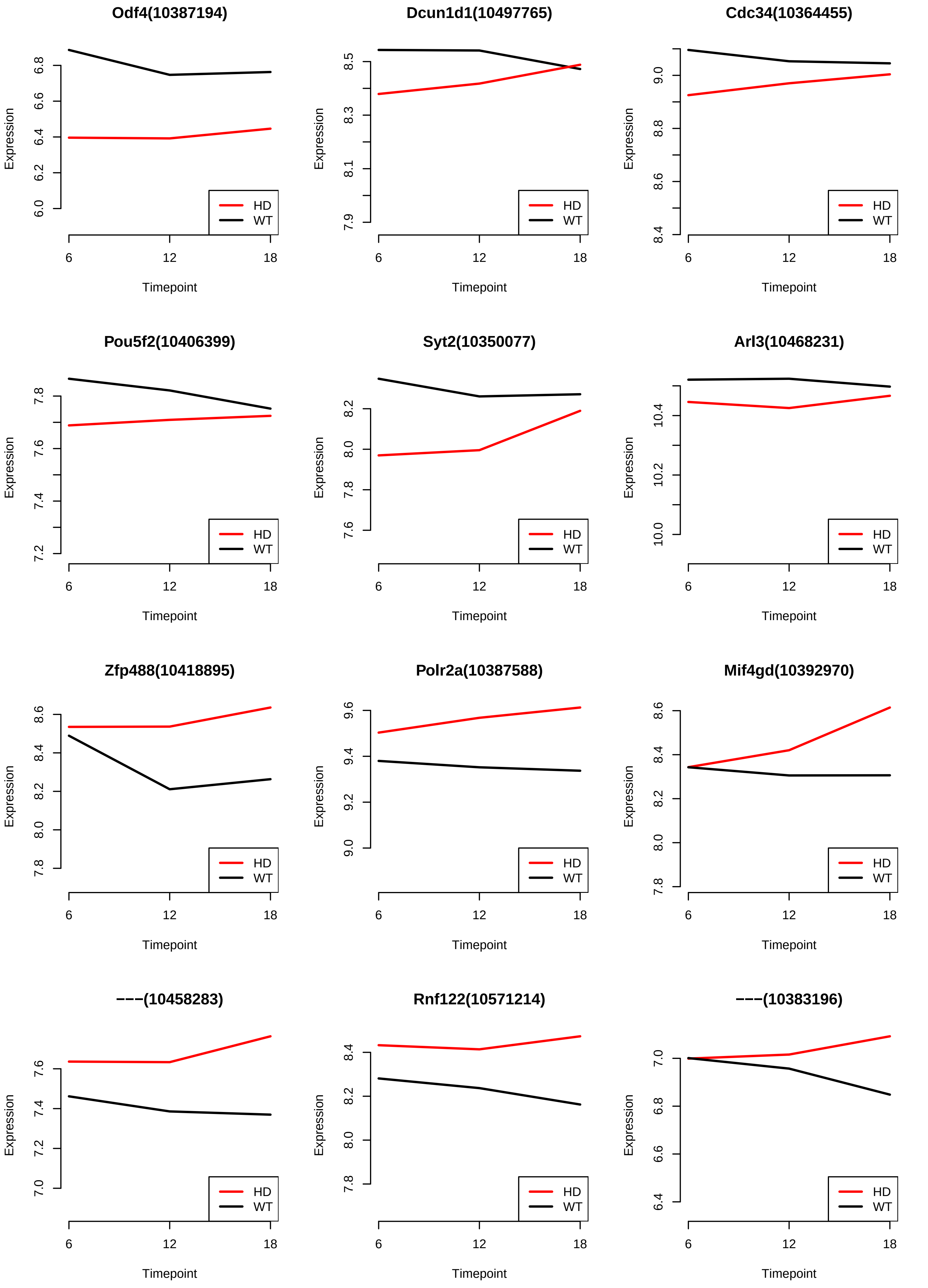
Odf4(10387194)
Dcun1d1(10497765)
Cdc34(10364455)
Pou5f2(10406399)
Syt2(10350077)
Arl3(10468231)
Zfp488(10418895)
Polr2a(10387588)
Mif4gd(10392970)
---(10458283)
Rnf122(10571214)
---(10383196)
Expression
Timepoint
6
12
18
HD
WT

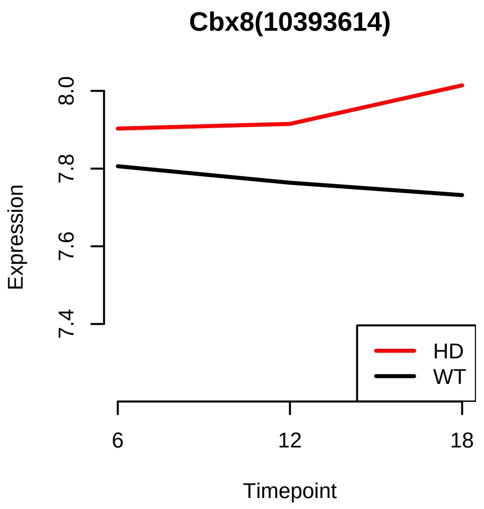

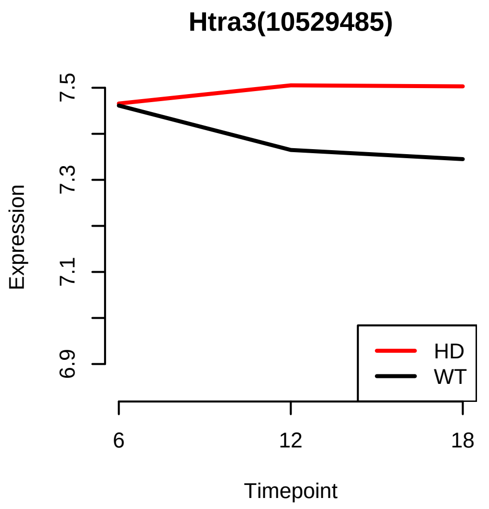

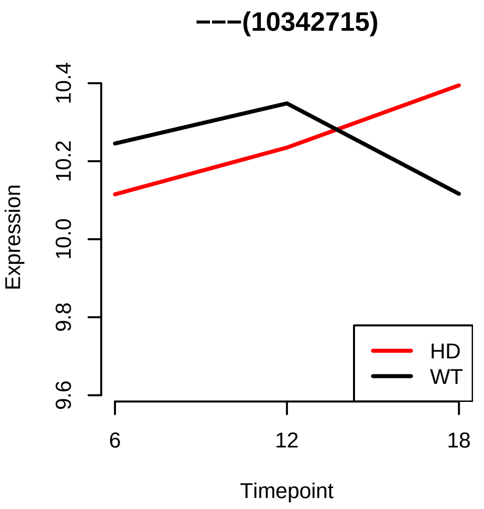

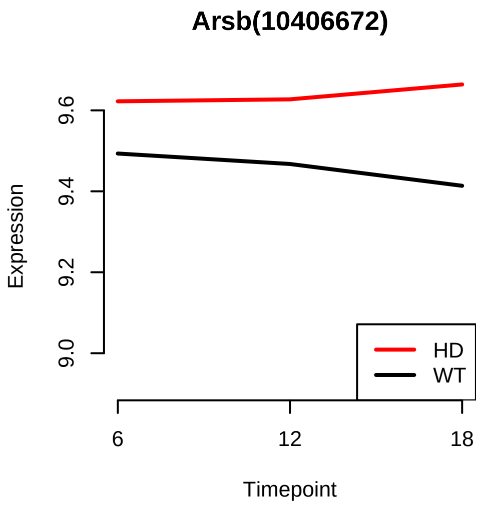

WT down
YAC128 up

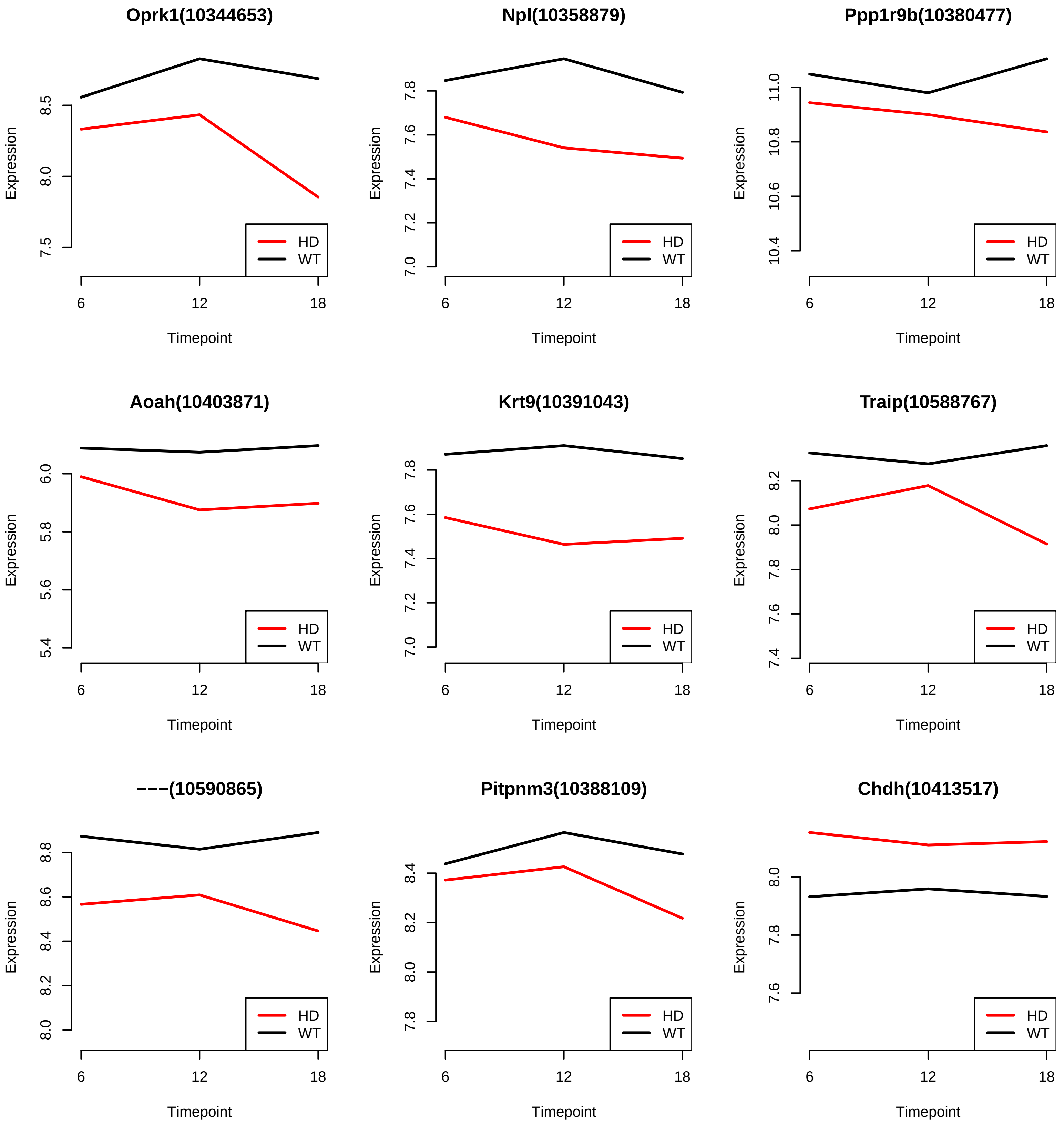

WT no change
YAC128 down

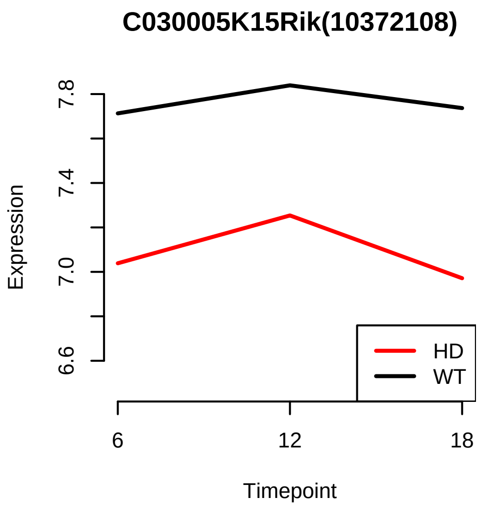

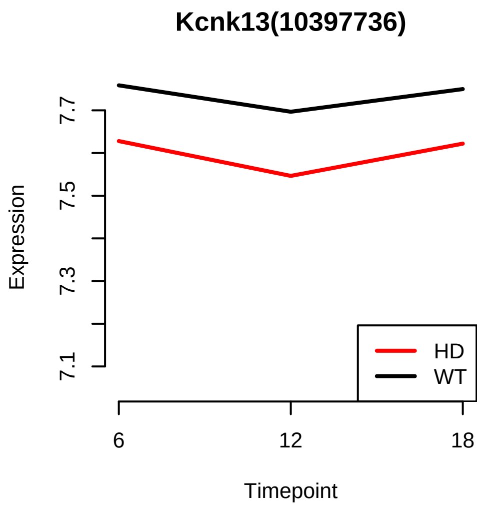

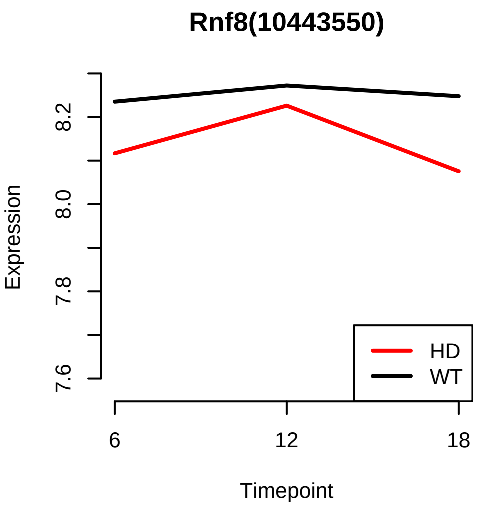

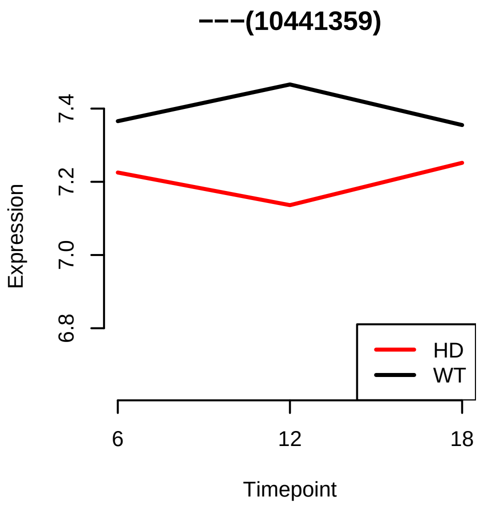

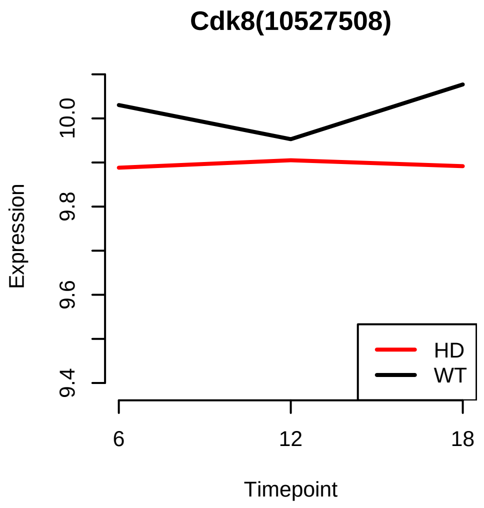

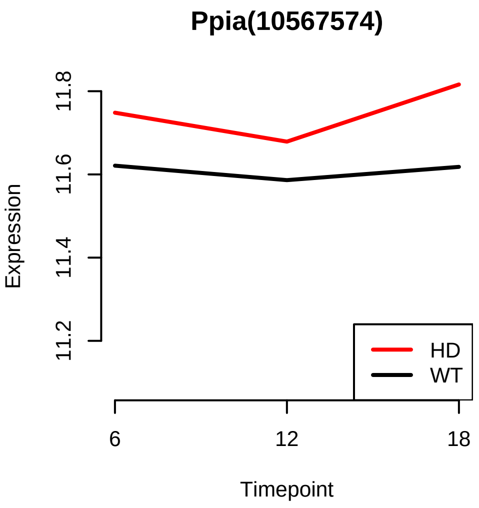

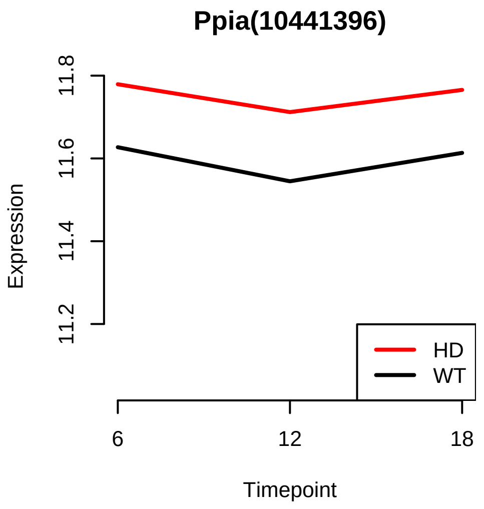

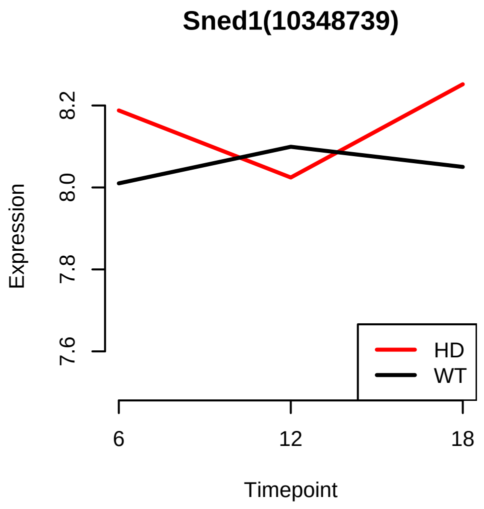

WT no change
YAC128 no
change

on next page:
WT no change
YAC128 up

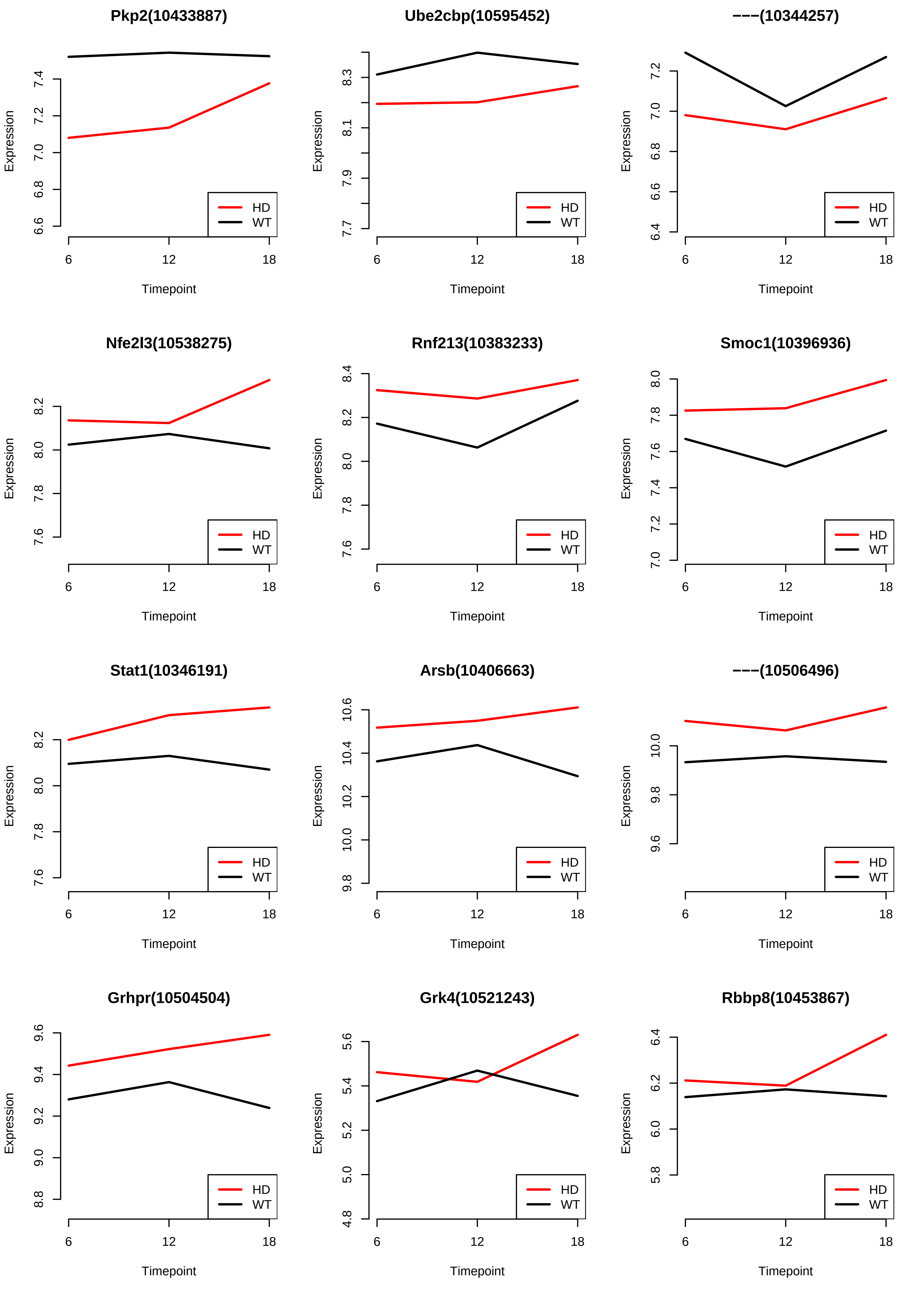
Pkp2(10433887)
Ube2cbp(10595452)
---(10344257)
Nfe2l3(10538275)
Rnf213(10383233)
Smoc1(10396936)
Stat1(10346191)
Arsb(10406663)
---(10506496)
Grhpr(10504504)
Grk4(10521243)
Rbbp8(10453867)
Expression
Timepoint
6
12
18
HD
WT

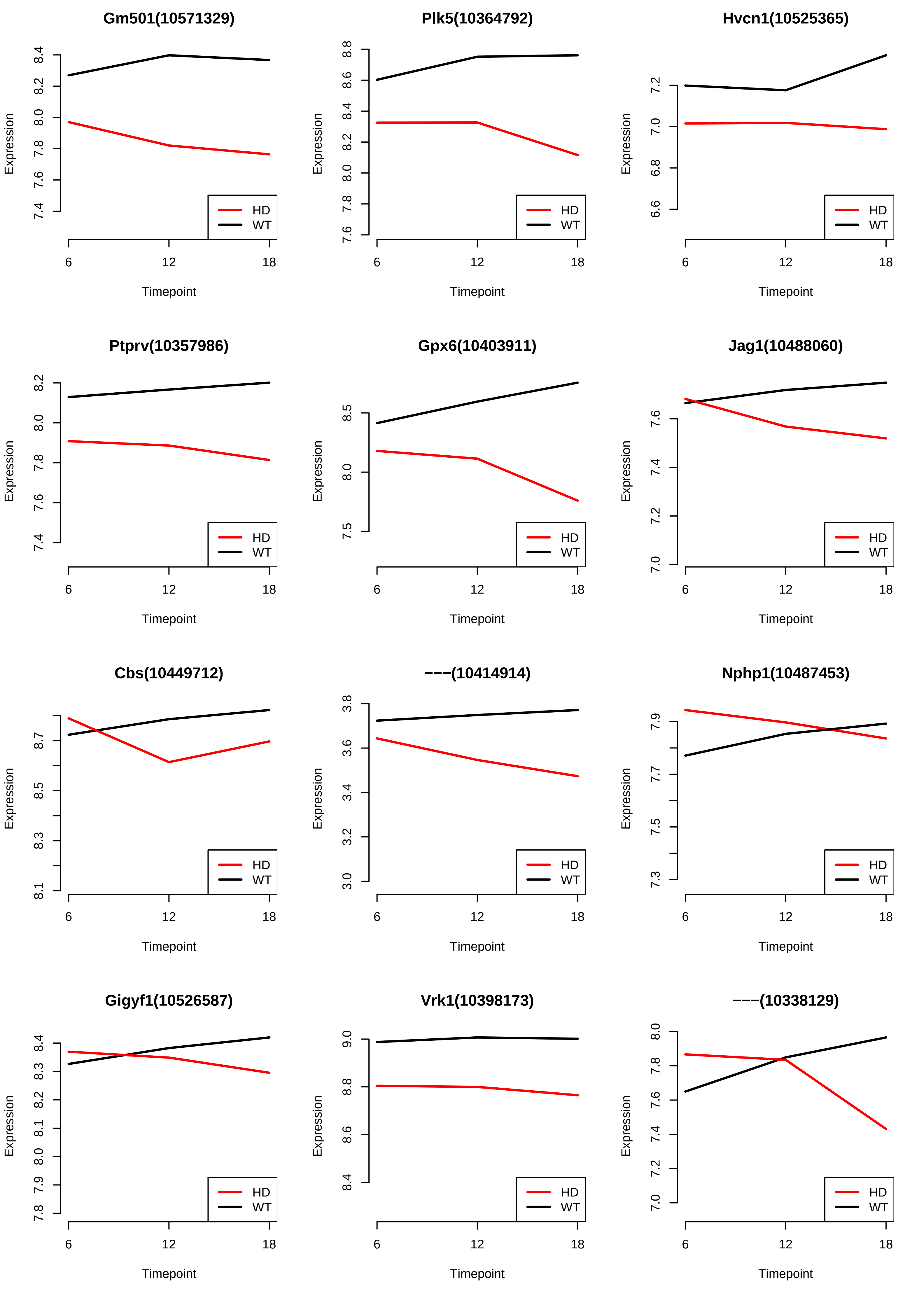
Gm501(10571329)
Plk5(10364792)
Hvcn1(10525365)
Ptprv(10357986)
Gpx6(10403911)
Jag1(10488060)
Cbs(10449712)
---(10414914)
Nphp1(10487453)
Gigyf1(10526587)
Vrk1(10398173)
---(10338129)
Expression
Timepoint
6
12
18
HD
WT

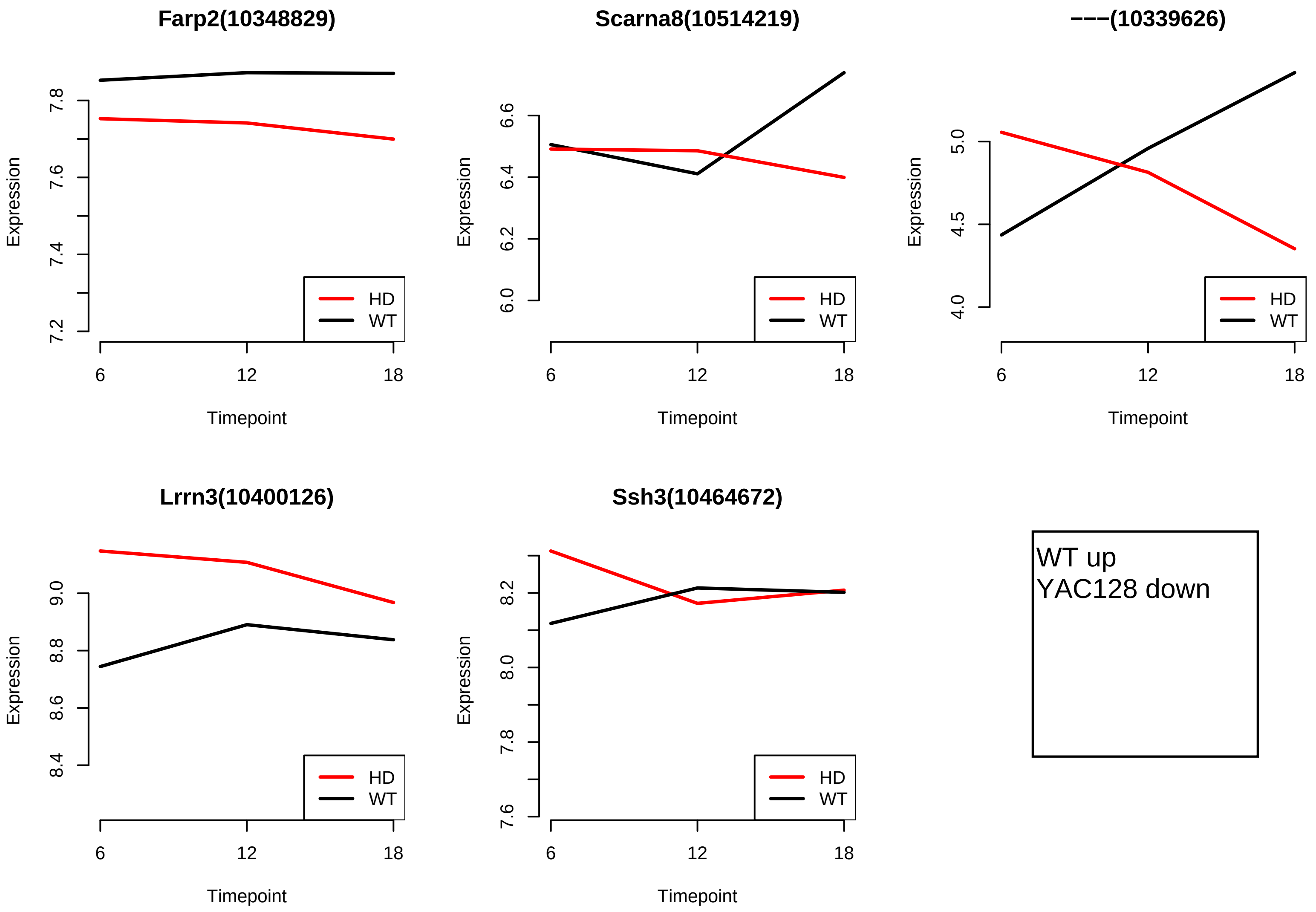
Farp2(10348829)
Scarna8(10514219)
---(10339626)
Lrrn3(10400126)
Ssh3(10464672)
Expression
Timepoint
6
12
18
HD
WT
7.2
7.4
7.6
7.8
6.0
6.2
6.4
6.6
4.0
4.5
5.0
8.4
8.6
8.8
9.0
7.6
7.8
8.0
8.2
WT up
YAC128 down

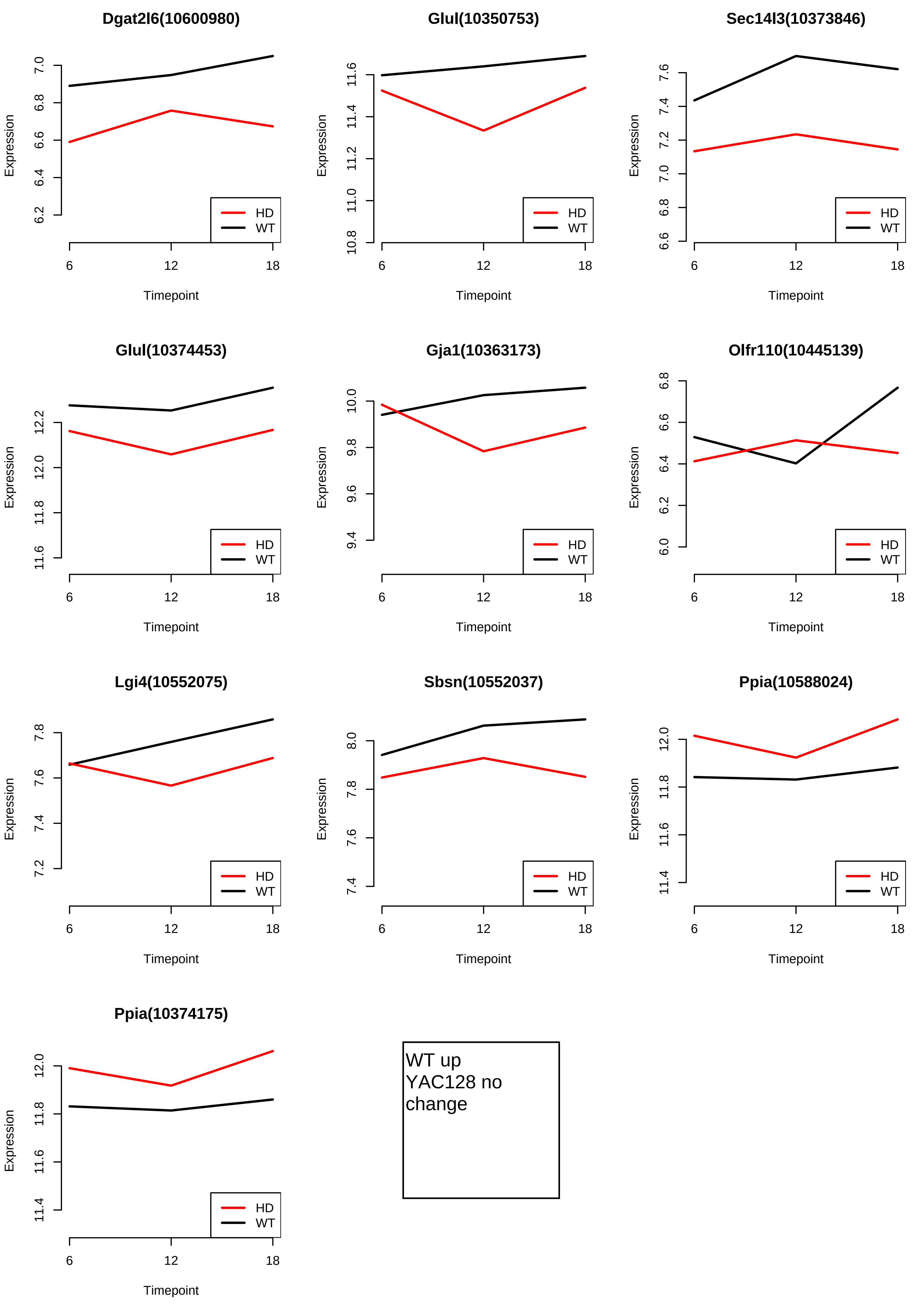
Dgat2l6(10600980)
Glul(10350753)
Sec14l3(10373846)
Glul(10374453)
Gja1(10363173)
Olfr110(10445139)
Lgi4(10552075)
Sbsn(10552037)
Ppia(10588024)
Ppia(10374175)
Expression
Timepoint
HD
WT
WT up
YAC128 no
change

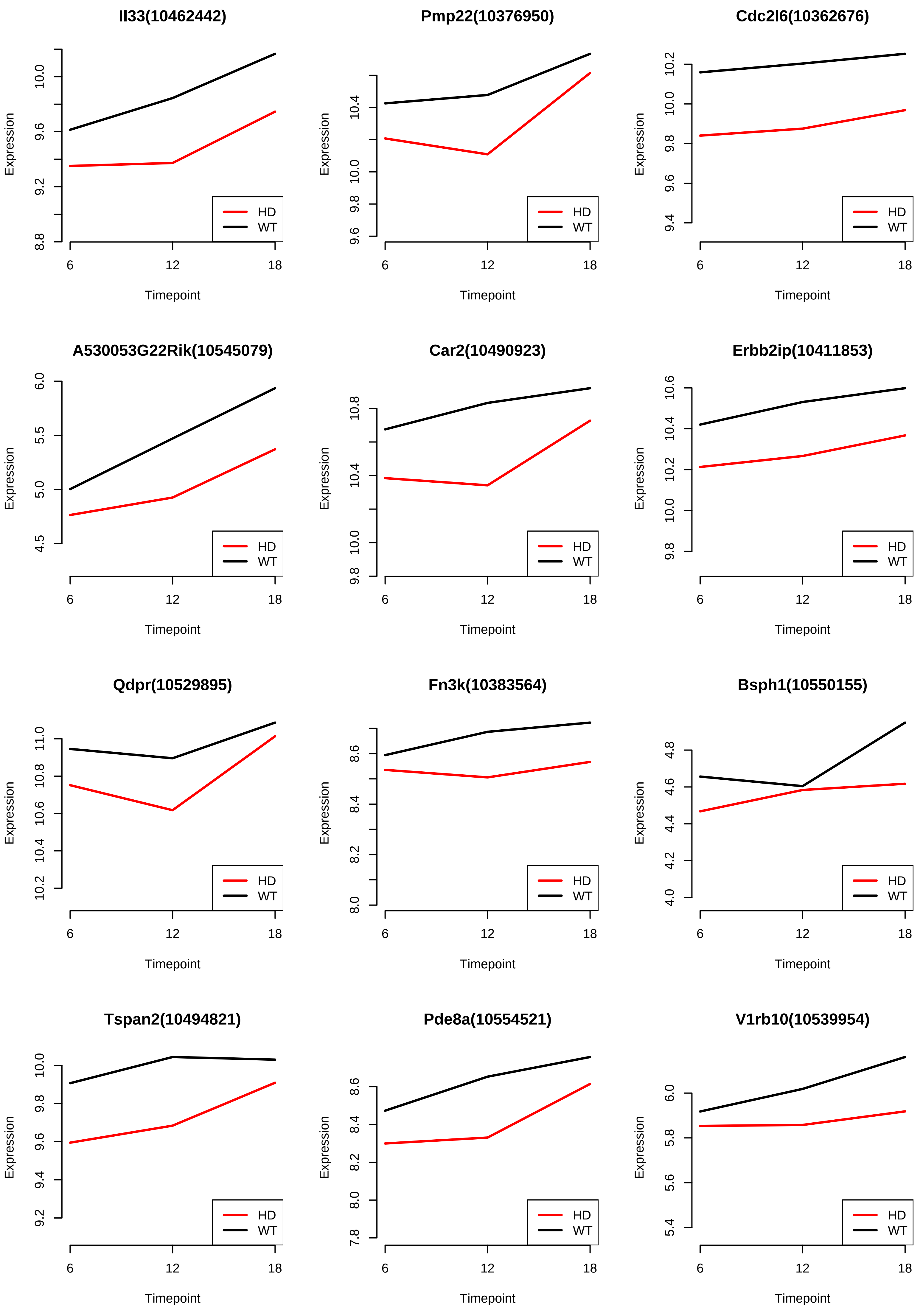
Il33(10462442)
Pmp22(10376950)
Cdc2l6(10362676)
A530053G22Rik(10545079)
Car2(10490923)
Erbb2ip(10411853)
Qdpr(10529895)
Fn3k(10383564)
Bsph1(10550155)
Tspan2(10494821)
Pde8a(10554521)
V1rb10(10539954)
Expression
Timepoint
6
12
18
HD
WT
8.8
9.2
9.6
10.0
9.6
9.8
10.0
10.4
9.4
9.6
9.8
10.0
10.2
4.5
5.0
5.5
6.0
9.8
10.0
10.4
10.8
9.8
10.0
10.2
10.4
10.6
10.2
10.4
10.6
10.8
11.0
8.0
8.2
8.4
8.6
4.0
4.2
4.4
4.6
4.8
9.2
9.4
9.6
9.8
10.0
7.8
8.0
8.2
8.4
8.6
5.4
5.6
5.8
6.0

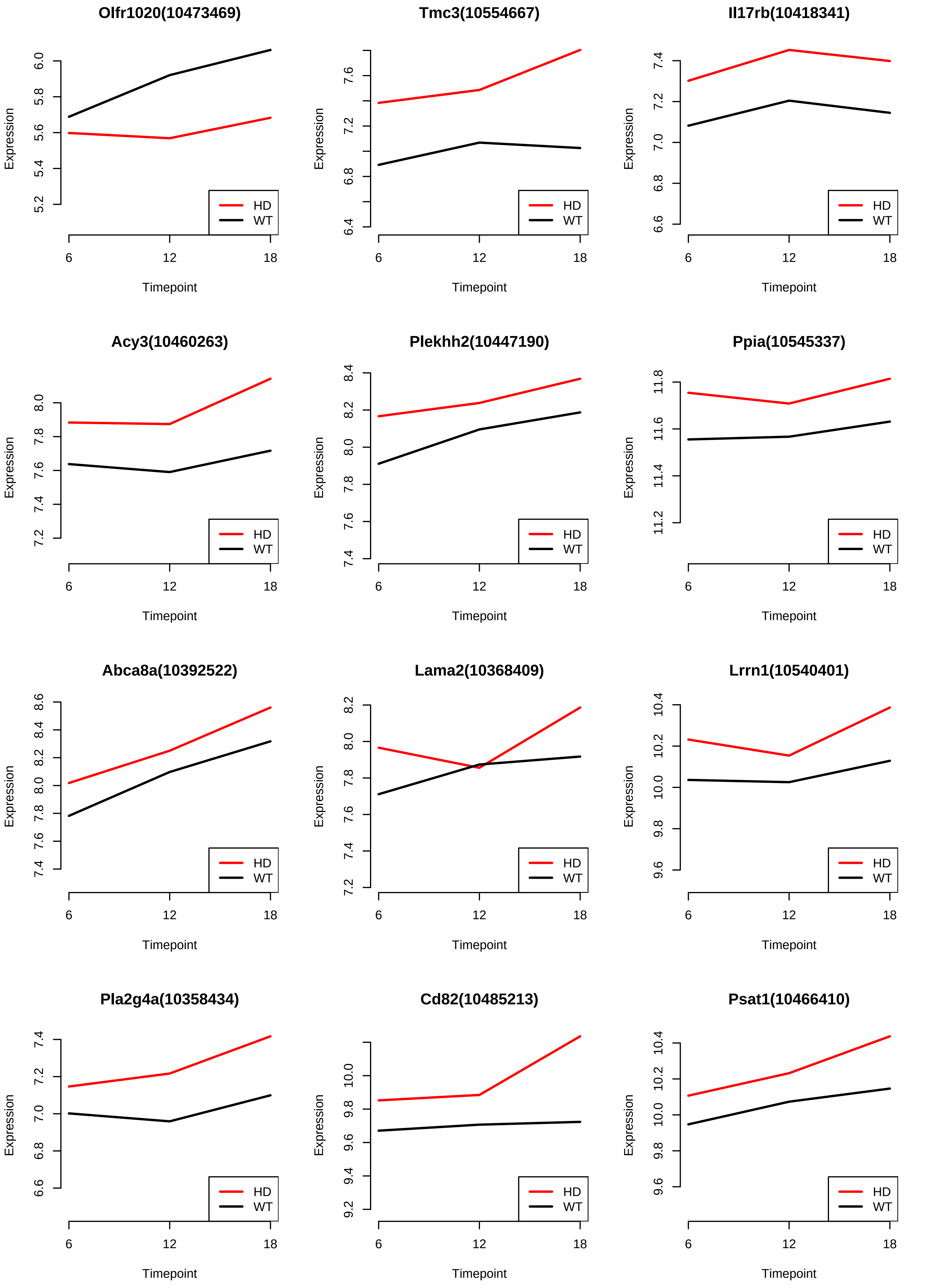

Olfr1020(10473469)
Expression
5.2
5.4
5.6
5.8
6.0
Timepoint
6
12
18
HD
WT
Tmc3(10554667)
Expression
6.4
6.8
7.2
7.6
Timepoint
6
12
18
HD
WT
Il17rb(10418341)
Expression
6.6
6.8
7.0
7.2
7.4
Timepoint
6
12
18
HD
WT
Acy3(10460263)
Expression
7.2
7.4
7.6
7.8
8.0
Timepoint
6
12
18
HD
WT
Plekhh2(10447190)
Expression
7.4
7.6
7.8
8.0
8.2
8.4
Timepoint
6
12
18
HD
WT
Ppia(10545337)
Expression
11.2
11.4
11.6
11.8
Timepoint
6
12
18
HD
WT
Abca8a(10392522)
Expression
7.4
7.6
7.8
8.0
8.2
8.4
8.6
Timepoint
6
12
18
HD
WT
Lama2(10368409)
Expression
7.2
7.4
7.6
7.8
8.0
8.2
Timepoint
6
12
18
HD
WT
Lrrn1(10540401)
Expression
9.6
9.8
10.0
10.2
10.4
Timepoint
6
12
18
HD
WT
Pla2g4a(10358434)
Expression
6.6
6.8
7.0
7.2
7.4
Timepoint
6
12
18
HD
WT
Cd82(10485213)
Expression
9.2
9.4
9.6
9.8
10.0
Timepoint
6
12
18
HD
WT
Psat1(10466410)
Expression
9.6
9.8
10.0
10.2
10.4
Timepoint
6
12
18
HD
WT

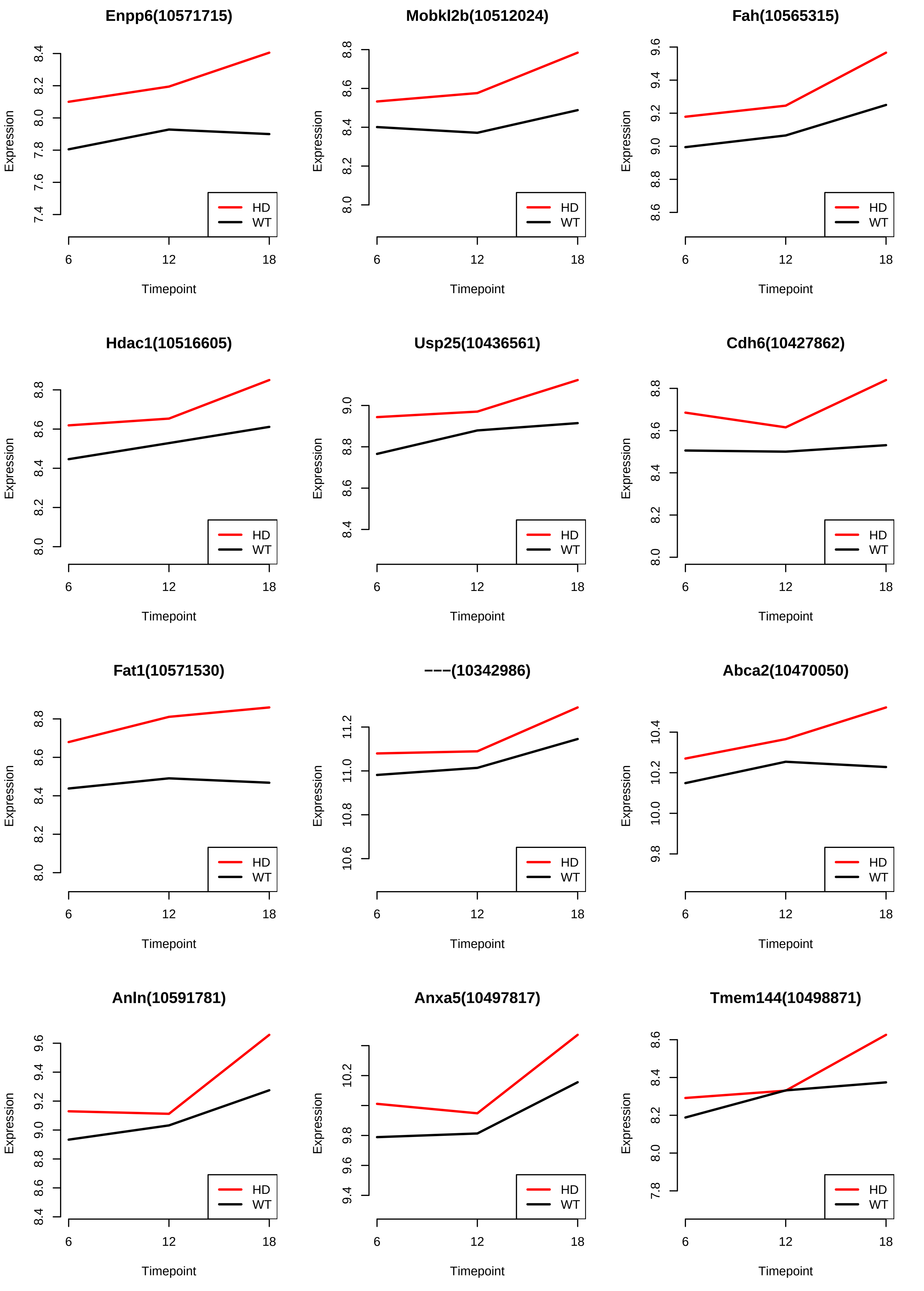
Enpp6(10571715)
Expression
7.4
7.6
7.8
8.0
8.2
8.4
HD
WT
6
12
18
Timepoint
Mobkl2b(10512024)
Expression
8.0
8.2
8.4
8.6
8.8
HD
WT
6
12
18
Timepoint
Fah(10565315)
Expression
8.6
8.8
9.0
9.2
9.4
9.6
HD
WT
6
12
18
Timepoint
Hdac1(10516605)
Expression
8.0
8.2
8.4
8.6
8.8
HD
WT
6
12
18
Timepoint
Usp25(10436561)
Expression
8.4
8.6
8.8
9.0
HD
WT
6
12
18
Timepoint
Cdh6(10427862)
Expression
8.0
8.2
8.4
8.6
8.8
HD
WT
6
12
18
Timepoint
Fat1(10571530)
Expression
8.0
8.2
8.4
8.6
8.8
HD
WT
6
12
18
Timepoint
---(10342986)
Expression
10.6
10.8
11.0
11.2
HD
WT
6
12
18
Timepoint
Abca2(10470050)
Expression
9.8
10.0
10.2
10.4
HD
WT
6
12
18
Timepoint
Anln(10591781)
Expression
8.4
8.6
8.8
9.0
9.2
9.4
9.6
HD
WT
6
12
18
Timepoint
Anxa5(10497817)
Expression
9.4
9.6
9.8
10.2
HD
WT
6
12
18
Timepoint
Tmem144(10498871)
Expression
7.8
8.0
8.2
8.4
8.6
HD
WT
6
12
18
Timepoint

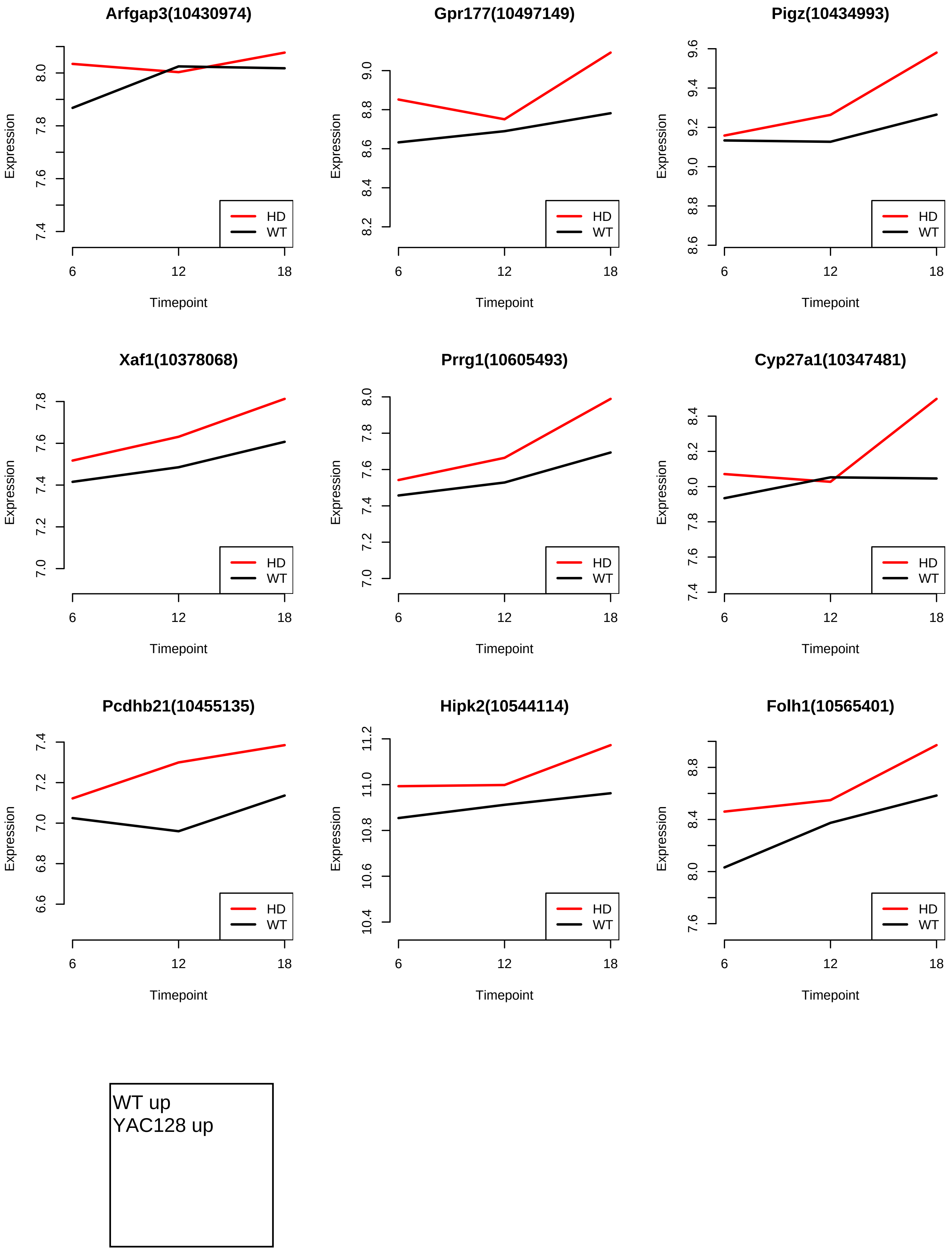
Arfgap3(10430974)
Gpr177(10497149)
Pigz(10434993)
Xaf1(10378068)
Prrg1(10605493)
Cyp27a1(10347481)
Pcdhb21(10455135)
Hipk2(10544114)
Folh1(10565401)
Expression
Timepoint
6
12
18
HD
WT
WT up
YAC128 up
