# Supplementary figures and images for: Similar striatal gene expression profiles in the striatum of the YAC128 and HdhQ150 mouse models of Huntington’s disease are not reflected in mutant Huntingtin inclusion prevalence
Source: BMC Genomics. 2015 Dec 21;16:1079. doi: 10.1186/s12864-015-2251-4 (PMC4687121; doi:10.1186/s12864-015-2251-4)

A

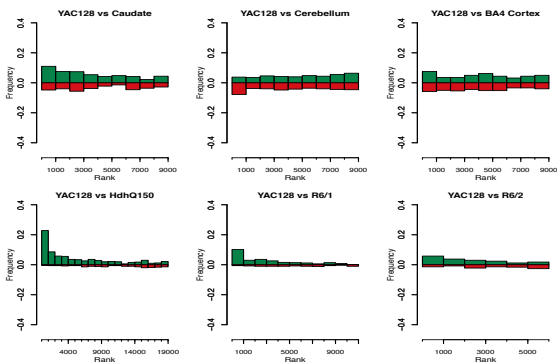

B

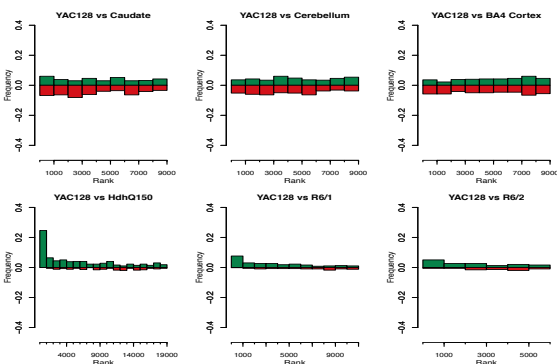

C

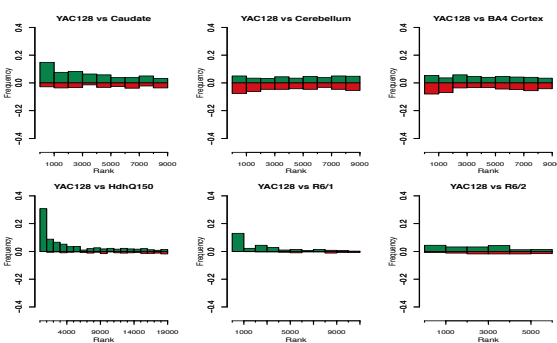

Supplement: Additional file 6: Figure S2. — Analysis of the overlap of changes in gene expression in the YAC128 striatum at A. 6 months of age B. 12 months of age and C. 18 months of age compared with other HD model mouse striata and human brain. Frequency represents the fraction of the top 200 HdhQ150 expression changes that map to a particular bin of ranked data (1000 genes per bin) in the other dataset, which is then split to identify concordant or discordant direction of expression change. A higher frequency of concordant (green) rather than discordant (red) in the first bins indicates a similarity between the YAC128 caudate and other model or human HD gene expression signature. (PDF 59 kb) [file 12864_2015_2251_MOESM6_ESM.pdf]
